# Re-examination of two diatom reference genomes using long-read sequencing

**DOI:** 10.1186/s12864-021-07666-3

**Published:** 2021-05-24

**Authors:** Gina V. Filloramo, Bruce A. Curtis, Emma Blanche, John M. Archibald

**Affiliations:** 1 Department of Biochemistry and Molecular Biology, Dalhousie University, PO Box 15000, Sir Charles Tupper Medical Building, 5850 College Street, Halifax, Nova Scotia B3H 4R2 Canada; 2 Centre for Comparative Genomics and Evolutionary Bioinformatics, Dalhousie University, Halifax, Nova Scotia Canada

**Keywords:** Diatom genomics, Oxford Nanopore long-read sequencing, Bionano optical mapping, Long-terminal repeat retrotransposons, *Phaeodactylum tricornutum*, *Thalassiosira pseudonana*

## Abstract

**Background:**

The marine diatoms *Thalassiosira pseudonana* and *Phaeodactylum tricornutum* are valuable model organisms for exploring the evolution, diversity and ecology of this important algal group. Their reference genomes, published in 2004 and 2008, respectively, were the product of traditional Sanger sequencing. In the case of *T. pseudonana*, optical restriction site mapping was employed to further clarify and contextualize chromosome-level scaffolds. While both genomes are considered highly accurate and reasonably contiguous, they still contain many unresolved regions and unordered/unlinked scaffolds.

**Results:**

We have used Oxford Nanopore Technologies long-read sequencing to update and validate the quality and contiguity of the *T. pseudonana* and *P. tricornutum* genomes. Fine-scale assessment of our long-read derived genome assemblies allowed us to resolve previously uncertain genomic regions, further characterize complex structural variation, and re-evaluate the repetitive DNA content of both genomes. We also identified 1862 previously undescribed genes in *T. pseudonana*. In *P. tricornutum*, we used transposable element detection software to identify 33 novel *copia*-type LTR-RT insertions, indicating ongoing activity and rapid expansion of this superfamily as the organism continues to be maintained in culture*.* Finally, Bionano optical mapping of *P. tricornutum* chromosomes was combined with long-read sequence data to explore the potential of long-read sequencing and optical mapping for resolving haplotypes*.*

**Conclusion:**

Despite its potential to yield highly contiguous scaffolds, long-read sequencing is not a panacea. Even for relatively small nuclear genomes such as those investigated herein, repetitive DNA sequences cause problems for current genome assembly algorithms. Determining whether a long-read derived genomic assembly is ‘better’ than one produced using traditional sequence data is not straightforward. Our revised reference genomes for *P. tricornutum* and *T. pseudonana* nevertheless provide additional insight into the structure and evolution of both genomes, thereby providing a more robust foundation for future diatom research.

**Supplementary Information:**

The online version contains supplementary material available at 10.1186/s12864-021-07666-3.

## Background

Diatoms (Bacillariophyta) are one of the most species-rich and successful photosynthetic eukaryotic groups on the planet, having evolved to occupy diverse ecological niches in marine, freshwater and even wet terrestrial habitats [1, 2]. Belonging to the stramenopiles, diatoms are thought to have originated approximately 250–190 million years ago (Mya); they contain red alga-type plastids that evolved via eukaryote-eukaryote endosymbiosis [3–5]. Diatoms are divided into two subdivisions that diverged 231–181 Mya: the Coscinodiscophytina, which includes the Coscinodiscophyceae (radial centric diatoms), and the Bacillariophytina, including the Mediophyceae (polar centric diatoms and radial centric Thalassiosirales) and the Bacillariophyceae (the pennate diatoms) [1, 6–10]. The important role of diatoms in global carbon fixation, primary productivity and ecosystem stability, as well as the complex evolution of their plastids, have placed these organisms at the forefront of ecological, biogeochemical and comparative genomic studies.

The first diatom nuclear genomes to be sequenced were those of the radial centric diatom *Thalassiosira pseudonana* (CCMP1335) [3] and the pennate diatom *Phaeodactylum tricornutum* (CCMP632) [11]*.* Both reference genomes were constructed using whole genome shotgun paired-end Sanger sequencing of small-, medium- and large-insert genomic libraries [3, 11]. The original version of the *T. pseudonana* haploid nuclear genome included 2170 contigs assembled into 1271 scaffolds totaling 35.6 megabase pairs (Mbp) [3]. Optical restriction site mapping further resolved 90% of the scaffolds to 24 chromosomes [3]. Subsequently, Bowler et al. [11] reassessed low quality and gap regions in the initial *T. pseudonana* genome and generated an improved 32.4 Mbp assembly that included 115 contigs assembled into 64 scaffolds representing the 24 chromosomes initially described by Armbrust et al. [3]. In comparison, the haploid 27.4 Mbp *P. tricornutum* genome assembly was comprised of 179 contigs assembled into 33 chromosome-sized scaffolds [11]. Both genome projects predicted protein-coding genes based in part on expressed sequence tag (EST) data, identifying species-specific genes as well as genes shared between the two species and among all stramenopiles [3, 11]. Despite the relatively recent divergence of the Mediophyceae and Bacillariophyceae (~ 172 Mya), the gene contents of these two diatom species are very different: only ~ 57% of *P. tricornutum* genes were found to be shared with *T. pseudonana*, suggesting that diatoms and their genomes have diversified at an unusually rapid rate [11].

The original *T. pseudonana* and *P. tricornutum* genome projects also shed light on the complex evolutionary history of diatoms. Both diatom genomes were found to be a mosaic of both heterotrophic host and algal endosymbiont genes, as well as apparently non-endosymbiotic, bacterial genes predicted to have been acquired via horizontal gene transfer (HGT) [3, 11–13]. Previous diatom genomic studies also demonstrated that transposable elements (TEs) are present in both genomes but are more prominent in *P. tricornutum* (8.4% of the *P. tricornutum* genome [14] vs. 1.9% in *T. pseudonana* [15]). The most abundant TEs in both diatoms are *Ty1*/*copia*-like long terminal repeat retrotransposons (LTR-RTs), which have been suggested to be significant drivers of diatom genome evolution, gene expression and adaptation to environmental changes [15–17]. Genomic restructuring and innovation resulting from LTR-RT activity, as well as the acquisition of genes from endosymbiotic and horizontal gene transfer, have no doubt contributed to the overwhelming success and diversity of diatoms across the globe.

Since the release of the *T. pseudonana* and *P. tricornutum* genomes, six additional diatom genomes representing both centric and pennate species have been published [18–25]. Some of the more recently released diatom genomes (e.g., *Cyclotella cryptica, Fragilariopsis cylindrus,* and *Seminavis robusta*) were generated using long-read sequencing [22, 24, 25]. As a so-called third-generation sequencing technology, long-read sequencers such as the Oxford Nanopore Technologies (ONT) MinION device, have the ability to generate ultra-long sequencing reads (10 kilobase pairs (Kbp) - 1 Mbp) derived from single molecules of native DNA [26]. Read lengths of several Kbp are advantageous in that they have the ability to span large repetitive or complex regions of DNA that are challenging to resolve with short-read [~ 300–500 base pairs (bp)] sequence data [27, 28]. Although long-read sequencing can yield highly contiguous genome assemblies, it is also notoriously error-prone (5–20% average error rate depending on the library preparation method, MinION chemistry and/or basecalling software) with most errors occurring as indels that have the potential to impact downstream analyses such as gene prediction [29–31]. To overcome the high per-base error rates associated with MinION sequencing, long-read assemblies are typically corrected or ‘polished’ using ONT raw signal data as well as high-quality, high-coverage Illumina short-read data [32]. By doing so, basecalling accuracy can improve up to 99.8% [33, 34]. The quality and contiguity of long-read derived genome assemblies can potentially be further improved using optical genome mapping and scaffolding, such as the Bionano Genomics platform [35] (for a detailed overview of the Bionano Genomics workflow, refer to the following resources: [36–39]).

Recent genome re-sequencing efforts have focused on organisms for which reference genomes were generated prior to the availability of next-generation and/or long-read sequencing methods (e.g., [25, 40–44]). Early reference genomes (like those of *P. tricornutum* and *T. pseudonana*) assembled from Sanger-based shotgun sequence data from mate paired libraries benefitted from low per-base error rates but were laboriously produced and limited by short-read lengths and low throughput [45, 46]. Consequently, genome assembly algorithms often failed to confidently resolve repeat sequences and/or complex structural variants, leading to highly fragmented reference genomes that lacked chromosome-scale context, underestimated repeat content and/or included misoriented contigs [47, 48]. The application of MinION long-read sequencing has improved the quality and completeness of existing reference genomes for both model and non-model organisms (e.g., *Arabidopsis thaliana, Caenorhabditis elegans, Cyclotella cryptica, Giardia intestinalis* WB, maize, *Nelumbo nucifera*) by drastically improving contig length and assembly contiguity, recovering ‘missing’ sequences, more accurately representing repeat content, and enhancing gene discovery and annotation [25, 40–44].

In this study, we used Oxford Nanopore long-read sequencing to produce updated versions of the *T. pseudonana* and *P. tricornutum* reference genomes*.* While the initial *T. pseudonana* and *P. tricornutum* reference genome sequences were meticulously generated using the most advanced sequencing strategies and assembly algorithms available at the time, both reference genomes contain a number of large gaps in the main scaffolds, as well as numerous shorter contigs that cannot be placed in a chromosomal context, and poorly understood chromosomal rearrangements and duplication events [3, 11]. We demonstrate the utility of long-read sequencing for validating the existing reference assemblies, identifying missing sequences and mis-assemblies in the original genomes, and determining the chromosomal context of previously unanchored contigs. We further explored our *T. pseudonana* long-read genome assembly by reassessing gene content for this species using published RNA-Seq data, identifying 1862 previously unreported genes. We also reassessed LTR-RT content for both diatom species, finding a much larger number of full-length, putatively active LTR-RT loci than previously described. Finally, we used Bionano genome mapping to further assess the *P. tricornutum* long-read assembly and determine whether long-read sequencing and optical mapping can serve as effective tools for the resolution of haplotypes in this and other species.

## Results and discussion

### Oxford Nanopore long-read sequencing

The nuclear genomes of *Phaeodactylum tricornutum* (CCMP632) and *Thalassiosira pseudonana* (CCMP1335) were de novo sequenced and assembled using long-read data generated on Oxford Nanopore’s MinION device (Fig. S1). For *P. tricornutum*, 986,604 reads (820,187 of which “passed” QC based on Albacore q-score binning) resulted in a total of 8.2 gigabase pairs (Gbp) of data (~300x coverage) with a mean read length of 8.3 Kbp, a mean read quality score (Q-score) of 8.5, and a read-length N50 of ~ 18.8 Kbp (Table 1, Table S1). For *T. pseudonana*, 701,596 reads were obtained (580,845 “passed”) totaling 7.5 Gbp of sequence (~230x coverage) with a mean read length of 10.6 Kbp, a Q-score of 9.9 and read-length N50 of ~ 20.1 Kbp (Table 1, Table S1). The reads were mapped to the existing diatom reference genomes to estimate nanopore read accuracy. For *P. tricornutum,* 76.8% of the unfiltered nanopore reads aligned to the reference genome with an average of 73.7% identity, while 76.6% of the unfiltered *T. pseudonana* long-read data aligned to the reference genome with an average identity of 71.5% (Table 1, Table S1).

**Table 1:** Raw read data summary for unfiltered, Albacore “passed” and filtered Oxford Nanopore long-read sequencing datasets for *Thalassiosira pseudonana* and *Phaeodactylum tricornutum*. The unfiltered data include all sequence reads, including passed (q-score>7) and failed (q-score<7) reads as determined by Albacore. The Albacore “pass” data include all reads with a quality-score >7. The filtered datasets for *T. pseudonana* and *P. tricornutum* included reads with read length ≥30 kb and ≥20 kb, respectively

		Unfiltered Data	Albacore “pass” Data	Filtered Data
*Phaeodactylum tricornutum*	Total bases (Gbp)	8.2	7.5	2.7
	No. of reads	986,604	820,187	84,445
	Mean read length (bp)	8,311.1	9,144.7	31,973.9
	Mean read quality	8.5	9.2	9.6
	Read length N50 (bp)	18,756	19,261	32,648
	Estimated genome coverage	~300x	~273x	~100x
	Percentage of reads mapped to JGI reference	76.8%	87.8%	92.6%
	Average percent identity of reads to JGI reference	73.7%		
*Thalassiosira pseudonana*	Total bases (Gbp)	7.5	7.0	1.8
	No. of reads	701,596	580,845	46,708
	Mean read length (bp)	10,611.8	12,029.7	37,942.3
	Mean read quality	9.9	10.8	10.9
	Read length N50 (bp)	20,088	20,514	37,303
	Estimated genome coverage	~230x	~215x	~50x
	Percentage of reads mapped to JGI reference	76.6%	89.1%	93.5%
	Average percent identity of reads to JGI reference	71.5%		

In order to produce high-quality genome assemblies for *T. pseudonana* and *P. tricornutum*, we curated sub-datasets of reads by filtering the original long-read datasets and selecting the highest quality reads of ≥20 Kbp and ≥ 30 Kbp for *P. tricornutum* and *T. pseudonana*, respectively, while maintaining at least 50x coverage of both genomes (based on the estimated genome sizes reported by Armbrust et al. [3] and Bowler et al. [11]). The read length cut-offs for each species were determined in part by considering the read length N50 of the filtered datasets as well as the minimum read-length necessary to span transposable elements (based on previously reported TE sequences by Maumus et al. [15]) and the estimated gap sizes in the original reference genomes [3, 11]. Filtering resulted in smaller but higher quality read datasets for both organisms, indicated by improved mean read length, mean Q-score and read length N50 metrics (Table 1, Table S1; Fig. S2). The filtered dataset for *P. tricornutum* included 8.6% (84,445 reads) of the original long-read dataset (986,604 reads); however, the mean read length improved over four-fold to 32.6 Kbp and the mean Q-score increased to 9.6 (Table 1, Table S1). The filtered *T. pseudonana* dataset included only 6.7% (46,708 reads) of the initial read dataset (701,596). The mean read length increased over three-fold (37.9 Kbp) while the Q-score increased to 10.9 (Table 1, Table S1). For both diatoms, the read length N50 increased from ~ 20 Kbp to over 30 Kbp (Table 1, Table S1). These curated datasets were used for subsequent de novo genome assembly.

### De novo genome assembly and analysis

De novo assemblies of the filtered datasets were produced using two dedicated long-read assemblers, Canu [49] and Flye [50, 51]. The raw *T. pseudonana* and *P. tricornutum* Canu and Flye assemblies suffered most noticeably from low percent identify to the reference genome and poor gene completeness (see below)—both symptoms of the high per-base error rate of nanopore sequencing (Table 2, Table S2). To correct mismatches and indels, the raw assemblies were first polished with long-read data using two rounds of Racon [52], followed by the complete Nanopolish [53] pipeline and at least ten rounds of Pilon [33], which uses Illumina-generated short-read data to correct false SNPs and erroneous insertions and deletions.

**Table 2:** Assembly statistics for the original reference genomes and de novo long-read derived genomes for *Thalassiosira pseudonana* and *Phaeodactylum tricornutum*. The mitochondrial and organellar genomes for both diatoms were assembled by Canu and Flye and were excluded from assembly statistical analyses. All Canu and Flye assemblies were corrected first by long-reads using Racon and Nanopolish followed by Illumina short-reads using Pilon (See Methods and Materials for more details). The BUSCO odb9 eukaryotic database (303 genes) was used to assess the different assemblies. The BUSCO scores are reported for the total gene completeness (C), complete single-copy (S), complete duplicated (D) and fragmented (F) orthologs

	Assembly	Total length (Mbp)	Read depth coverage	No. contigs	Largest contig (Mbp)	Contig N50 (Mbp)	Contig L50	No. scaffolds	Largest scaffold (Mbp)	Scaffold N50 (Mbp)	Scaffold L50	G+C content (%)	% identity to reference	BUSCO Complete-ness	ALE score
*Phaeodactylum tricornutum*	Reference (Bowler et al. 2008)	27.4	9.6x	179	n/a	0.42	20	88^a^	2.53	0.95	11	48.8	n/a	C:82.5% *S:80.2%* *D:2.3%* F:5.9%	n/a
	Canu	57.0	40x	291	2.51	0.25	43	n/a	n/a	n/a	n/a	48.7	99.3	C 85.4% *S:33.3%* *D:52.1%* F:3.0%	-734,959,595
	Flye	33.5	72x	196	1.66	0.36	24	n/a	n/a	n/a	n/a	48.7	99.1	C:80.9% *S:71.0%* *D:9.9%* F:4.6%	-781,367,384
	Canu-Bionano hybrid	66.8	n/a	n/a	n/a	n/a	n/a	219^b^	2.78	1.06	n/a	n/a	n/a	n/a	n/a
*Thalassiosira pseudonana*	Reference (Armbrust et al. 2004, Bowler et al. 2008)	32.4	n/a	115	n/a	1.27	8	64^c^	3.04	1.99	7	46.9	n/a	C:81.2% *S:79.2%* *D:2.0%* F:5.3%	n/a
	Canu	47.3	40x	222	2.77	0.98	14	n/a	n/a	n/a	n/a	46.9	99.4	C:79.2% *S:59.7% D:19.5%* F:6.6	-1,238,092,187
	Flye	33.8	48x	52	2.76	1.38	8	n/a	n/a	n/a	n/a	47.0	99.4	C:80.6% *S:78.9%* *D:1.7%* F:5.6%	-1,047,071,217

^a^The number of scaffolds reflects the 33 chromosome-level scaffolds and 55 unplaced, smaller contigs.

^b^The number of scaffolds for the Canu-Bionano hybrid includes both the 49 scaffolds that were assembled from the 138 long-read contigs that met minimum length requirement (≥150 kb) for Bionano optical map anchoring and the 155 unanchored contigs <150 kb.

^c^The number of scaffolds reflects the 27 chromosome-level scaffolds and 37 unplaced, smaller contigs.

Overall, our polishing approach resulted in progressively improved measures of genome quality and completeness (contiguity, percent identity to the reference genome, error rate, gene content and Assembly Likelihood Evaluation (ALE) score; Table 2, Table S2). Comparison of the final polished Canu and Flye long-read assemblies to the previously published reference genomes yielded average sequence identities of ~ 99% for both *T. pseudonana* and *P. tricornutum* (Table 2, Table S2), a noticeable improvement over the initial 71–73% average mapping identities (Table 1).

The final polished Canu and Flye assemblies for *T. pseudonana* included a minimum of 40x read depth coverage, with a genome size for the final polished Flye assembly of 33.8 Mbp; this is consistent with the reference genome [3, 11] (Table 2, Table S2). In stark contrast, the polished Canu-derived assembly for *T. pseudonana* was 47.3 Mbp, which is over 10 Mbp larger than the reference genome size (Table 2, Table S2). The Flye assembly was more contiguous (52 contigs) than both the existing reference genome (115 contigs) and the Canu assembly (222 contigs; Table 2, Table S2). Compared to the Canu assembly, the Flye assembly had a longer contig N50 (1.38 Mbp vs 0.98 Mbp) and a lower contig L50 (8 vs 14; Table 2, Table S2).

Genome assembly trends were similar for *P. tricornutum* in that the final polished long-read assembly generated with Flye yielded a smaller genome size and lower number of contigs compared to Canu (Table 2, Table S2). While the *P. tricornutum* Flye assembly was somewhat larger than the existing reference genome (33.4 Mbp versus 27.4 Mbp), the Canu assembly was 66.8 Mbp, more than double the expected genome size. Despite using reads ≥30 Kbp, both the Flye (196 contigs) and Canu assemblies (291 contigs) were less contiguous than the existing *P. tricornutum* reference assembly (179 contigs). Unsurprisingly, the more contiguous Flye assembly had better contig N50 and L50 statistics, although the largest contig generated for *P. tricornutum* was produced by the Canu assembly (2.51 Mbp vs 1.66 Mbp with Flye; Table 2, Table S2).

In order to select the best overall assembly, the Canu and Flye assemblies were evaluated based on a combination of traditional assembly metrics, statistical analysis tools and gene completeness assessments. First, the accuracy of our de novo genome assemblies was assessed using the ALE pipeline [54]. This statistical tool uses a Bayesian framework to detect synthetic errors in genome assemblies and calculate the likelihood that an assembly is correct given the raw data underlying it. The overall ALE score is calculated based on four ‘sub-scores’: (i) the placement score, which assesses how well each mapped read corresponds to the assembly, (ii) the insert score, which evaluates the expected paired-end read length in the assembly, (iii) the depth score, which measures sequencing depth consistency across the assembly, and (iv) the k-mer score, which uses k-mer frequency to calculate the assembly likelihood independent of the read data [54]. Combined, these four values provide a more objective measure for comparing assemblies based on the same read dataset but produced by different assembly tools; the assembly with the highest ALE score is statistically the best and thus most likely to be correct. For *T. pseudonana*, the more contiguous Flye assembly with better L50 and N50 values was determined to be the ‘best’ genome assembly (Table 2, Table S2). However, our data show that contiguity may not always be the best indicator of the highest quality assembly. While the Flye assembly for *P. tricornutum* was the most contiguous, it was statistically worse than the more fragmented Canu assembly based on ALE (Table 2, Table S2).

**Fig. 1:**
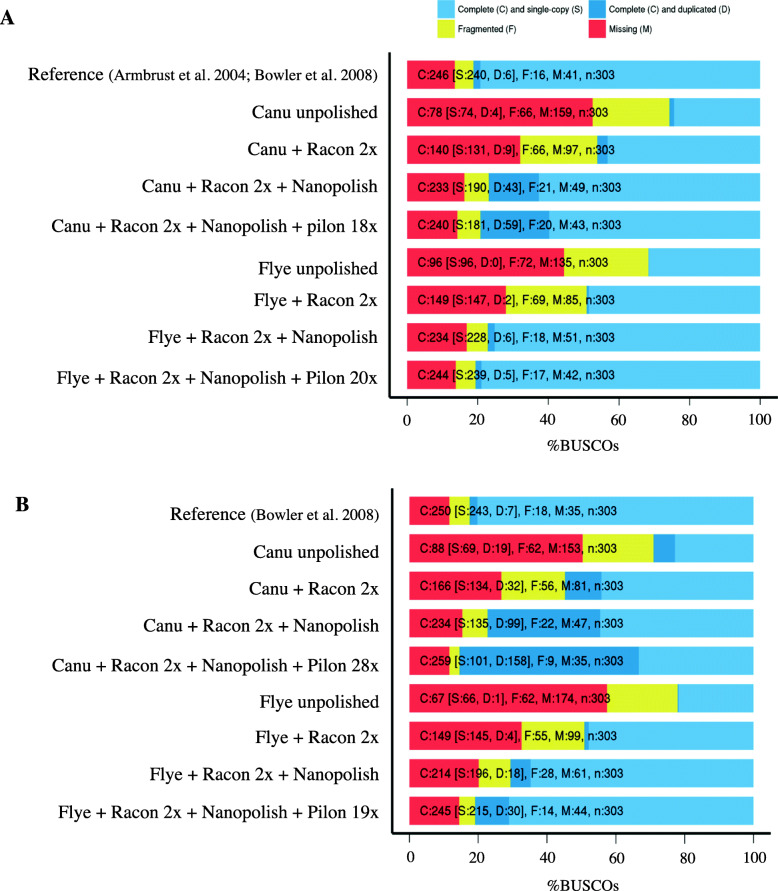
Genome completeness using single-copy orthologs (BUSCO eukaryota_odb9 database) was assessed for the *Thalassiosira pseudonana* (**a**) and *Phaeodactylum tricornutum* (**b**) reference genomes as well as the unpolished and polished versions of the Canu and Flye de novo assemblies for both diatom species. Note that the BUSCO analysis was performed after each step of the polishing pipeline which included two rounds of Racon, followed by Nanopolish and finally, multiple iterations of Pilon

To assess genome completeness, orthologs from a set of conserved single-copy eukaryotic genes were identified for each genome using BUSCO v3.0.2 [55]. For *T. pseudonana*, BUSCO completeness for the Flye assembly and the published reference genome assembly was similar [Flye = 80.6%, 244 out of 303 total genes present; reference = 81.2%, 246 out of 303 total genes], with the majority (~ 79.0%) of the genes in both the Flye and reference assemblies existing as complete single copies (Table 2, Table S2; Fig. 1a). The Canu assembly was deemed similarly complete (79.2%, 240 out of 303 genes), although it contained fewer complete single copy genes (59.7%) and a larger proportion of complete duplicated genes (19.5%) than the *T. pseudonana* Flye and reference genome assemblies (Table 2, Fig. 1a).

For *P. tricornutum*, BUSCO completeness values for our Flye assembly and the reference genome were similar: 80.9% (245 out of 303 total genes) and 82.5% (250 out of 303 total genes), respectively (Table 2, Table S2; Fig. 1b). While our analyses detected mostly single copy complete genes (80.2%, 243 genes) and few complete duplicated genes (2.3%, 7 genes) for the original reference genome, the proportion of duplicated complete genes in the Flye assembly increased 4-fold (9.9%, 30 genes). Our Canu assembly had the highest BUSCO score (85.4%, 259 out of 303 genes), although it recovered fewer single copy genes (33.3%, 100 out of 303 genes) and a disproportionate number of complete duplicated genes (52.1%, 158 out of 303 genes) relative to the *P. tricornutum* Flye and reference assemblies (Table 2, Table S2; Fig. 1b). The large number of duplicated genes detected in the *P. tricornutum* Canu assembly raised the possibility that the assembly algorithm either resolved both haplotypes of the diploid genome (see below) or revealed large segmental duplications that were collapsed in the reference assembly.

We ultimately settled on the *T. pseudonana* Flye and *P. tricornutum* Canu assemblies, which were finalized with our full polishing pipeline, as the ‘best’ overall de novo assemblies for downstream analyses. Their robustness and completeness reflect the benefits of combining long-reads for the generation of long contigs with the accuracy of Illumina short-read data. The short-reads are needed to correct indel errors in the nanopore data, as indicated by the dramatically improved estimates of genome completeness and ALE scores with each iteration of our polishing pipeline (Fig. 1; Table 2, Table S2). Although the final *T. pseudonana* Flye assembly achieved greater contiguity than the original reference genome (which included 37 unplaced contigs), the *P. tricornutum* Canu assembly was over three times more fragmented than expected (Table 2, Table S2). The *T. pseudonana* Canu assembly was also significantly more fragmented than that produced with Flye.

The fragmented nature of the Canu assemblies for both diatom genomes is a consequence of the different way that the two assemblers handle allelic diversity and repetitive genomic content. While Canu is a more conservative assembler that is capable of resolving highly divergent haplotypes, low-complexity and highly repetitive areas [49], Flye may be prone to merging alleles and collapsing repetitive content, often resulting in more artifactually contiguous assemblies [51]. After our analyses were completed, a newer version of Flye (v2.4) was released that is less prone to collapsing repeats and alleles; it produced an assembly for *P. tricornutum* that was slightly larger in size (39 Mbp), more fragmented (433 contigs) and had a smaller contig N50 (0.15 Mbp) than our initial Flye (v2.3) assembly. Although further analysis is required, the more fragmented nature of the Flye v2.4 assembly suggests that less repetitive content was collapsed and/or fewer alternative haplotypes were merged. It is worth noting that although Canu generates more fragmented assemblies that are less useful for inferring genomic structure and organization, Flye assemblies (v2.3 and older) are also imperfect in that they are more likely to exclude biologically real and potentially important genetic information. The abundance of LTR-RTs in the *P. tricornutum* genome (see below) likely confounded the Canu assembly algorithm and contributed to the fragmented nature of the final assembly. In the *P. tricornutum* genome, LTR-RT insertions often occur in just one of the haplotypes. Canu’s conservative algorithm likely detected discrepancies between allele-specific reads that were otherwise the same and did not merge those reads into a single contig. Instead, the algorithm separated those reads (those with the LTR-RT insertion and those without) and produced two distinct contigs (i.e., alternative haplotypes), which resulted in double the expected genome size based on the reference [11]. That said, the Canu assembly is likely closer to reality than the reference or Flye v2.3 assemblies because it captures more of the complexities intrinsic to the *P. tricornutum* genome. In the case of *T. pseudonana*, further exploration of the Canu assembly is needed in order to determine if its fragmented nature is the result of greater LTR-RT content than previously recognized in the reference genome [3, 11]. For the time being, if we assume that LTR-RTs in *T. pseudonana* follow a similar pattern of haplotype-specific insertions, the decreased number of LTR-RT insertions in *T. pseudonana* compared to *P. tricornutum* (see below) likely resulted in less ‘haplotype phasing’ by the Canu algorithm and as a result, the *T. pseudonana* Canu assembly was not as inflated relative to the reference [3, 11].

### Long-read sequencing resolves outstanding issues in existing diatom reference genomes

#### Resolution of telomeres and unlinked chromosome scaffolds

Our long-read assemblies resolved some of the unanswered questions posed by the *T. pseudonana* and *P. tricornutum* reference genomes, including unresolved telomeres and unplaced scaffolds. The 27.4 Mbp *P. tricornutum* reference genome was predicted to contain 33 chromosomes based on the assembly of 33 scaffolds (87.9 Kbp to 2.5 Mbp) [11]. Out of the 33 chromosome-level scaffolds, 12 scaffolds achieved telomere-to-telomere resolution, 16 scaffolds contained one telomere, and five scaffolds lacked both telomeres. None of the contigs in our Canu long-read assembly contained telomeres at both ends, although 58 of these contigs have a telomere at one end. Bionano optical mapping (see below) assigned 32 Canu contigs with single telomeres to 29 Bionano-Canu hybrid chromosome-level scaffolds (out of 49 total hybrid scaffolds), resulting in three hybrid scaffolds with telomeres at both ends, and 26 hybrid scaffolds with a telomere at one end. Mapping our Canu contigs to the reference genome scaffolds indicated that 34 telomeres on the reference scaffolds were also present on the ends of the homologous Canu contigs. In some cases, telomeres were present on our Canu contigs but not their homologous counterparts in the reference scaffolds. While the *P. tricornutum* reference assembly has one telomere each for chromosomes 18 and 29, we were unable to uncover telomere sequence for the homologous Canu contigs. Our long-read sequencing combined with Bionano optical mapping suggests that the *P. tricornutum* genome contains at least 29 chromosomes. This estimate was further supported by pulsed-field gel electrophoresis (PFGE), which resolved at least 29 chromosomes ranging from ~ 480 Kbp to ~ 3.0 Mbp in size (Fig. S3). Additional chromosomes in the *P. tricornutum* genome may have been overlooked in our PFGE observations owing to the co-migration of multiple, similarly sized chromosomes.

The *T. pseudonana* reference genome was predicted to contain at least 24 chromosomes (297 Kbp to 3.04 Mbp), which were represented by six genome scaffolds with telomeres at both ends, 17 scaffolds with a telomere at only one end, and four scaffolds without telomeres at either end [3, 11]. Without scaffolding via optical mapping, our Flye long-read assembly did not achieve the same level of completion as the original reference. Comprised of 52 contigs, the Flye assembly contained only a single fully resolved telomere-to-telomere chromosome. That contig was homologous (99.6% identity) to reference chromosome 3, which has a telomere at one end. Single telomeres were resolved for 25 of the remaining Flye contigs, which, when mapped to the reference scaffolds, validated the resolution of the majority of the ‘single-telomere’ reference scaffolds. So, while our Flye assembly did not resolve chromosome-level contigs, it did map well to the more complete scaffolds of the reference genome [3, 11]. In some cases, the Flye contigs were able to resolve one or more telomeres where a reference chromosome only resolved one or none (i.e., Flye contigs included telomere sequence flanking the homologous region to the reference scaffold).

Based on optical restriction site mapping, the *T. pseudonana* reference genome identified two reference scaffolds as representative of chromosome 11 and two scaffolds corresponding to chromosome 16 [3, 11]. Telomere sequence was identified at one end of each of those *T. pseudonana* scaffolds (‘chr11a’, ‘chr11b’, ‘chr16a’, and ‘chr16b’); however, the two respective scaffolds could not be definitively linked to form two authentic chromosomes [3, 11]. In the case of ‘chr16a’ & ‘chr16b’, this was because the scaffolds were separated by repetitive sequence that was too long to be resolved by the length of a fosmid insert. Similarly, optical mapping attributed three *T. pseudonana* scaffolds (‘chr19a’, ‘chr19b’, and ‘chr19c’) to a single chromosome but sufficient nucleotide sequence data to demonstrate their connection was lacking. Mapping our Flye contigs to the reference genome allowed us to resolve the missing nucleotide data linking these fragmented reference chromosomes together, thereby validating three more complete chromosomes for *T. pseudonana*.

#### Resolution of unplaced scaffolds and gap filling with long-read data

The *P. tricornutum* and *T. pseudonana* reference genomes both include substantial amounts of sequence data that could not be placed in a larger chromosomal context at the time of publication. These unlinked scaffolds, termed “bottom drawer” (scaffolds prefixed as ‘Bd’), were predicted either to fall within unresolved gaps on the main chromosome-level scaffolds or to represent alternative haplotypes [3, 11]. The original *P. tricornutum* genome included 55 unlinked scaffolds 450 bp to 293 Kbp in size, while the *T. pseudonana* genome included 37 such scaffolds (2282 bp to 138 Kbp). Using long-read sequencing, we placed 30 out of 37 and 38 out of 55 *T. pseudonana* and *P. tricornutum* “bottom drawer” scaffolds, respectively (Table S3). This was achieved by manually identifying “bottom drawer” scaffolds with homology to contigs in our assemblies and bridging the reference genome gaps with our long-read-derived contigs.

The *P. tricornutum* and *T. pseudonana* reference genomes include 69 (~ 0.33 Mbp) and 18 (~ 0.10 Mbp) gap regions on the main chromosome scaffolds, respectively. We used local alignments between the reference chromosomes and their homologous long-read derived contigs to identify regions where our contigs spanned gaps in the reference chromosomes. In doing so, we filled in 13 gaps in the main scaffolds for *T. pseudonana* and 18 gaps for *P. tricornutum* (Table S4). We also assessed the gaps in the “bottom drawer” scaffolds for each diatom reference genome. Out of 31 (~ 53 Kbp) gaps for *T. pseudonana,* we resolved 12 (Table S4). For *P. tricornutum*, the 20 gaps (~ 97 Kbp) in the “bottom drawer” scaffolds could not be resolved as none of these scaffolds showed obvious homology to our Canu contigs. In total, by using long-read data to resolve the unlinked scaffolds and gaps associated with the reference assemblies, we were able to integrate 0.10 Mbp (*T. pseudonana*) and 0.49 Mbp (*P. tricornutum*) of additional sequence data into our assemblies relative to the original reference genomes.

#### Detection of structural variation

**Fig. 2:**
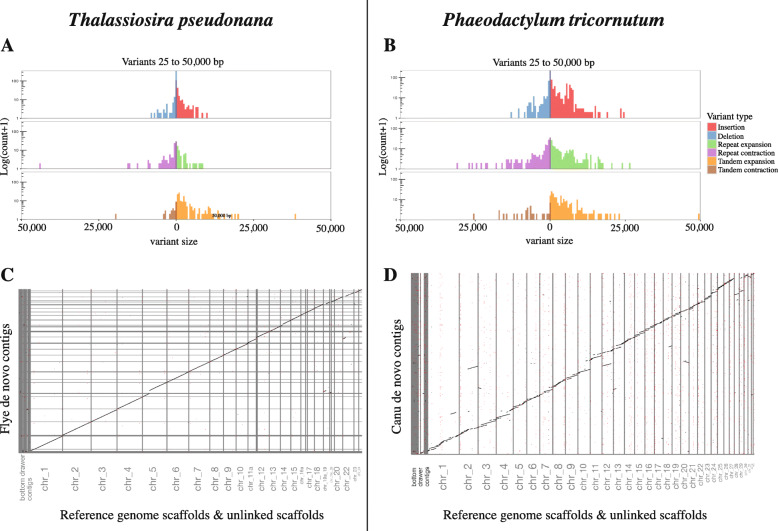
Assemblytics output plots showing six classes of structural variants between the *Thalassiosira pseudonana* reference genome and the final polished de novo long-read Flye assembly (**a**) and the *Phaeodactylum tricornutum* reference genome and the final polished de novo long-read Canu assembly (**b**). Dot plots comparing the *Thalassiosira pseudonana* reference chromosome-level scaffolds and unanchored contigs to the Flye assembly contigs (**c**) and the *Phaeodactylum tricornutum* chromosome-level scaffolds to the Canu assembly contigs (**d**) were also generated by Assemblytics

To assess small (< 50 bp) and large (> 50 bp) structural variation between the reference and long-read diatom genomes, we used Assemblytics [56] which detects and catalogs variants based on whole genome alignments generated by MUMmer. We found 1.20 Mbp of variants between the *T. pseudonana* Flye assembly and the original reference, with insertions and tandem expansions contributing to 58% of the total size variation (Fig. 2; Table S5). A total of 4.68 Mbp of structural variation was detected between our Canu *P. tricornutum* genome and the reference (Fig. 2; Table S5). Over 1.75 Mbp of that difference (624 variants in total) were attributed to insertions, with the majority (~ 1.12 Mbp) 4000–10,000 bp in size (Fig. 2). When variants in that size range were extracted and compared to a local database of diatom long-terminal repeat retrotransposons, 84% (157 out of 187 variants) were found to be CoDi LTR-RTs. Further investigation of all 1569 variants reported for the Canu *P. tricornutum* genome identified 25.5% (400 variants) as LTR-RTs, versus only 2.3% (21 out of 935 total variants) in *T. pseudonana* (a complete analysis of LTR-RTs is described below). A case-by-case investigation of the possible biological significance of these structural variations is beyond the scope of the present study but is certainly warranted.

#### Resolution of ribosomal RNA operons

Due to their multi-copy, homogeneous nature, nuclear ribosomal RNA (rRNA) operons are notoriously difficult to assemble using traditional sequence data; they thus serve as a useful test of the potential for long-read sequencing to improve genome assembly. To that end, the reference and polished long-read diatom genome assemblies were assessed for copies of the complete rRNA operon (18S, ITS1, 5.8S, ITS2, 28S). Whereas a single complete rRNA operon was detected on scaffold chromosome 17 in the *T. pseudonana* reference genome, our Flye assembly contained a single 733,359 bp contig (Flye contig3, which is homologous to reference scaffold chromosome 17) containing five complete tandem rRNA operon copies (Table 3). The average length of each complete rRNA operon was 5826.8 bp with an average of 4521.8 bp between each operon (Table 3). The five complete operons have an average identity of 99.6%. Two partial rRNA copies (1742 bp & 793 bp) were detected on the unlinked ‘bottom drawer’ reference scaffold Bd36x69, while the five complete rRNA copies on Flye contig3 were followed by a truncated (5538 bp) copy that was missing ~ 300 bp from the 28S portion of the operon.

**Table 3:** Summary of complete ribosomal operon (rRNA) statistics for *Phaeodactylum tricornutum* and *Thalassiosira pseudonana*

	*Phaeodactylum tricornutum*	*Thalassiosira pseudonana*
Number of complete tandem rRNA copies per contig	2 (contig 2792-chr7)	5 (contig3-chr17)
	5 (contig74-chr13)	
Average complete rRNA length	5,935.6 bp	5,826.8 bp
Average length of sequence between rRNA copies on same contig	15,611 bp (contig 2792-chr7)	4,521.8
	8,058 bp (contig74-chr13)	
Percent identity between copies on same contig	99.9 (contig 2792-chr7)	99.6-99.7
	99.9 [99.8-100] (contig74-chr13)	
Percent identity between copies on different contigs	99.5 [96.5-100]	n/a
Average illumina read depth at rRNA loci (avg read depth across genome)	82.5x (66x)	638x (148x)

To assess whether the tandem rRNA copies in *T. pseudonana* were mis-assemblies, we mapped our long-read data to the de novo Flye assembly. We detected multiple examples of single MinION reads that spanned all five rRNA copies located on Flye contig3, suggesting that the rRNA tandem array assembled by Flye was biologically accurate. However, the average Illumina read depth at those five rRNA loci was over four times the average read depth for the rest of the genome (638x vs. 148x) indicating that the *T. pseudonana* genome contains additional rRNA loci that were collapsed by the Flye assembly algorithm. Our detection of multiple rRNA copies at the end of Flye contig3, which is homologous to reference chromosome 17, is consistent with previous assessments of rRNA repeats for *T. pseudonana.* The initial version of the reference assembly for *T. pseudonana* reported a cluster of ~ 35 rRNA copies on chromosome 17; however, the assembler that was used to generate the second version of the *T. pseudonana* genome seems to have collapsed those repeats into a single rRNA locus on chromosome 17 [3, 11].

In the case of *P. tricornutum*, we identified a complete rRNA operon (5043 bp) on scaffold chromosome 13 of the reference genome as well as a partial operon (766 bp) on scaffold chromosome 7. In our long-read-derived assembly, we detected two Canu contigs with complete tandem rRNA copies (5935.6 bp average length) – two rRNA copies (99.9% identical) on contig2792 (homologous to reference scaffold chromosome 7) and five copies (99.9% average identity) on contig74 (homologous to reference scaffold chromosome 13; Table 3). Two partial rRNA copies (766 bp & 205 bp) were also detected on contig2792. Single MinION reads were found to span the two complete rRNA copies on contig2792, while multiple MinION reads were identified as spanning the five tandem rRNA copies on contig74. The *P. tricornutum* raw long-read data thus support the rRNA arrays detected on Canu contig2792 and contig74 as biologically authentic and not the result of mis-assemblies.

In contrast to *T. pseudonana,* the average Illumina read depth at the *P. tricornutum* Canu assembly rRNA loci was relatively similar to the average read depth across the entire genome (82.5x vs. 66x), suggesting that the long-read data capture the total number of rRNA loci in the genome as one would expect the read coverage to be a multiple of the average genomic coverage (e.g., 132x) if there were other copies of the rRNA operon that had been collapsed into this area. To assess if failure to resolve tandem rRNA arrays in the original reference genomes was a symptom of the assembly process collapsing highly repetitive genomic regions, we mapped the raw sequence data produced by Bowler et al. [11] to the intergenic spacer region (IGS) resolved in our Canu assembly. Those raw reads mapped to the IGS regions for both contig74 and contig2792 with an average read depth of ~ 7.0x (average read depth for entire contig = 7.0x) and 5.9x (average read depth for entire contig = 9.1x), respectively*.* This suggests that the tandem rRNA copies were indeed present in the reference data, but those regions were collapsed by the assembly algorithm. All things considered, our long-read assemblies provide a more accurate picture of the ribosomal RNA operon organization in the *T. pseudonana* and *P. tricornutum* genomes*.*

### De novo gene prediction and annotation for *Thalassiosira pseudonana*

Rastogi et al. [14] recently used RNA-Seq and traditional EST data to re-annotate the *P. tricornutum* genome, identifying 12,233 genes versus the 10,402 genes originally reported in the reference genome [11]. To our knowledge there have been no attempts to reinvestigate the gene content of *T. pseudonana* since Bowler et al. [11] predicted 11,673 genes in the nuclear genome, (~ 4000 of which were supported by EST data) based on their improvements to the initial reference assembly [3]. Our polished *T. pseudonana* Flye assembly served as the foundation for the gene comparison and re-discovery analyses below.

Comparison of our *T. pseudonana* Flye long-read assembly to the complete set of proteins predicted for the reference resulted in the identification of 99.9% of the previously reported genes; only eight genes out of 11,673 were not detected*.* Long-read mapping against both the reference and Flye assemblies identified long-reads that supported the presence of those eight genes in the reference sequence as well as long-reads that authenticated their absence in our Flye assembly. It is possible that each of those genes occurs at a single locus in the genome and is represented by only a single allele, resulting in two distinct haplotypes at a given locus. While the reference assembly resolved the haplotype version containing the allele, the Flye assembly resolved the alternate haplotype in which the allele has been lost.

Exploration of potentially ‘new’ gene content in *T. pseudonana* was performed using the Flye long-read assembly and a newly assembled transcriptome. Four RNA-Seq datasets previously published by Goldman et al. [57] were downloaded from NCBI and assembled into 22,600 transcripts corresponding to 10,383 protein coding genes. The assembled transcriptome was mapped against our *T. pseudonana* Flye genome for an overall alignment completeness of 95.3%. When the reference protein coding genes were compared against the new transcriptome, a total of 344 reference genes were not detected at the nucleotide level. In comparison, when the transcriptome was compared against the reference dataset of protein coding genes for *T. pseudonana*, ~ 2500 out of the 22,600 transcripts were not recovered (e-value = 1e-15), even though ~ 560 of the ~ 2500 transcripts were identified as being homologous to other diatom sequences in the NCBI protein database (e-value ≤1e-05) (mostly *Thalassiosira oceanica*; see below).

Comparison of the newly assembled, RNA-seq-based *T. pseudonana* transcriptome against the reference and Flye assemblies indicated a very small proportion of missing genes. Out of 22,600 transcripts (corresponds to 10,383 protein coding genes), only 57 did not have hits against the reference genome, versus 52 transcripts that did not have hits against the Flye de novo assembly. A total of 37 transcripts lacking hits were shared between the reference and Flye genomes and likely correspond to poorly assembled transcripts or contamination. The 20 transcripts missing from the reference genome and 15 transcripts missing from the Flye assembly likely correspond to genes located in missing genomic regions in each assembly.

We used the *T. pseudonana* transcriptome and long-read assembly to carry out a de novo gene prediction, resulting in a protein coding dataset of 16,491 genes, substantially larger than the 11,673 genes reported for the reference [3, 11]. Of the newly predicted genes, 13,805 (83.7%) were found to have high similarity (≥70% amino acid identity) to previously reported *T. pseudonana* genes (Fig. S4). Out of the remaining 2686 predicted genes (16%), 1971 had no match against the Armbrust et al. (2004) reference genome, while 715 had only a weak match (i.e., percent identity ≤70%), suggesting that those genes represent paralogs to recognized *T. pseudonana* genes (Fig. S4).

When the 2686 ‘new’ *T. pseudonana* gene sequences were compared to the NCBI protein database, 2010 genes had hits (<1e-03; Fig. S4). While 148 genes were most similar to transposon genes (transposon-related genes are not included in the reference protein coding gene set), the remaining 1862 genes were found to be most similar to genes identified in other diatom species (Fig. S4). Notably, 1042 of these genes were most similar to genes in *Thalassiosira oceanica* (data which were not available when the reference genome was published), suggesting that these 1862 genes are authentic, newly recognized *T. pseudonana* genes and not artefacts of the gene finding process. A blastp analysis against the NCBI protein database indicated that 1189 genes out of the 1862 newly predicted genes for *T. pseudonana* had homology to genes with known functions (not including genes associated with transposons) in other species. 676 genes did not have obvious homologs in the NCBI protein database and thus require further investigation to determine if they represent *T. pseudonana-*specific genes or are artefacts (e.g., due to intron retention in RNA-Seq data). 67 ‘new’ genes mapped to the “gap-resolved” regions (see above) of the *T. pseudonana* Flye assembly. Out of those 67 genes, 33 genes (49.2%) were among the 2686 genes without a blast hit showing ≤70% identity to the reference genome. While three of the 33 genes were identified as being transposons, the remaining 30 genes were previously unidentified in *T. pseudonana*.

Out of 11,673 genes predicted in the original *T. pseudonana* reference genome, 3900 were inferred to be specific to this genome and another 1407 were deemed diatom-specific [3, 11]. These numbers are based on comparison of the protein coding gene datasets for *T. pseudonana* and *P. tricornutum,* which were the only diatoms datasets available at that time. Since then, genomes and transcriptomes have been sequenced from a variety of additional diatom orders and genera, allowing for a more comprehensive and accurate assessment of species-specific and diatom-specific gene content. Comparison of our *T. pseudonana* proteome to protein datasets for seven other diatoms identified 3731 orthologous groups shared among the eight diatom species (Table 4). A total of 7512 (45.6%) *T. pseudonana* genes predicted in our study were assigned to these groups; 5136 genes were inferred to be diatom-specific, 1959 as shared with other stramenopile lineages (e.g., oomycetes, Blastocystidae, Pelagophyceae) and 1082 as having strong similarity to bacteria (predominantly Proteobacteria) as determined by subsequent PLAST analyses against the NCBI protein database. The *T. pseudonana* genes with a strong affinity to bacteria were not investigated further, although they could represent instances of HGT, as inferred by previous studies [11, 13].

**Table 4:** Orthologous group (OG) statistics for eight diatom genomes

Diatom species	Total protein coding genes	Proteins classified into OGs	Proteins not classified into OGs	OGs shared among all diatom species	Proteins in diatom-shared OGs
*Fragilariopsis cylindrus*	18,111	14,312	3,799	3,731	6,741
*Fistulifera solaris*	20,429	17,899	2,530		10,693
*Pseudo-nitzschia multiseries*	19,703	14,123	5,580		6,266
*Pseudo-nitzschia multistrata*	12,039	10,675	1,364		5,726
*Phaeodactylum tricornutum*	12,178	10,278	1,900		5,886
*Synedra acus*	27,337	17,403	9,934		9,326
*Thalassiosira oceanica*	34,642	16,486	18,156		8,781
*Thalassiosira pseudonana*	16,491	13,799	2,692		7,512

A total of 2692 *T. pseudonana* proteins were not assigned to orthologous groups (16.3% in total) and PLAST assessment against the NCBI protein database (e-value 1e-10, query coverage ≥70%, ≥40% identity) identified 2502 genes that were likely *T. pseudonana*-specific genes/proteins (Table 4). The remaining 190 genes showed obvious homology to other diatom sequences (predominantly *T. oceanica*). Out of the *T. pseudonana*-specific proteins, 716 were among the putative novel genes identified here-in.

The discovery of 1862 previously unreported genes in *T. pseudonana* was unexpected– it enhances our understanding of gene content for this species and provides a framework for consideration of which of its genes are ‘species-specific’ and ‘diatom shared’. The number of genes *T. pseudonana* shares with other diatoms will no doubt continue to grow as more genomes are sequenced.

### Bionano optical mapping of the *Phaeodactylum tricornutum* genome

Ploidy assessment of the de novo *P. tricornutum* Canu genome assembly is consistent with previous suggestions that *P. tricornutum* is a diploid organism (Fig. S5) [11, 58–61]. As noted above, PFGE (Fig. S3) supports the existence of at least 29 chromosomes (~ 480 Kbp to ~ 3.0 Mbp in size) totaling ~ 30–32 Mbp, which is roughly consistent with the number of chromosomes (33) and genome size (27.4 Mbp) reported for the reference. As noted above, our Canu long-read assembly was roughly double the expected genome size, supporting the separation of reads into two contigs representing different alleles. Bionano optical mapping was performed in an attempt to more accurately resolve both *P. tricornutum* haplotypes.

The Canu assembly was used to select the direct labeling enzyme DLE-1 as the best enzyme for achieving the recommended labeling density of 8–25 sites per 100 Kbp required for optimal resolution (DLE1 recognition site: CTTAAG, labeling density 7.501/100 Kbp). The Bionano system generated 4,760,428 virtually labeled molecules that were filtered (molecules ≥100 Kbp) for a total of 1,055,998 molecules (average length 252.6 Kbp) totaling 267 Gbp. Only Canu contigs greater than 150 Kbp (138 of 293 contigs, 71.8% of total assembly) were scaffolded onto the de novo Bionano physical consensus maps. Hybrid scaffolding produced 49 super-scaffolds (128 Kbp − 2.78 Mbp) totaling 50.6 Mbp (Table S6). When combined with the 155 contigs that were too small to be anchored to the physical consensus maps (28.2% of the Canu assembly), the total genome size increased to 66.8 Mbp. The N50 of the Bionano-Canu hybrid assembly was found to be 1.06 Mbp, representing a 4.2-fold increase when compared to the Canu assembly alone (Table 2; Table S6). The resulting 49 super-scaffolds included 9.5 Mbp of gaps (23 bp - 1.0 Mbp) with the majority of gaps (86%) being ≤300 Kbp in length.

Our Bionano data resolved multiple super-scaffolds that are homologous to the same regions of the reference chromosomes, supporting the separation of the Canu contigs and scaffolds into two copies. As the haploid *P. tricornutum* reference genome contains 33 chromosomes (12 scaffolds with telomeres at both ends), we anticipated resolution of 66 total haplotypes. The presence of only 49 super-scaffolds indicates that the Bionano-Canu hybrid assembly is missing 17 haplotype representative scaffolds. The 49 super-scaffolds were classified as either a “full-length haplotype” (i.e., super-scaffolds aligned to their homologous reference chromosome sequences across their entire length; 19 scaffolds in total), a “partial haplotype” (i.e., the super-scaffold aligned to only part of its homologous reference chromosome; 17 scaffolds), a “mis-assembled haplotype” (i.e., portions of a super-scaffold aligned to more than one reference chromosome; nine scaffolds) or an “unresolved haplotype” (i.e., the super-scaffold could not confidently be resolved to a reference chromosome; four scaffolds; Table S6).

When the super-scaffolds were mapped against the *P. tricornutum* reference chromosomes, we were only able to resolve full-length haplotypes for four reference chromosomes (chr1, chr8, chr16, and chr26, eight super-scaffolds in total; Fig. S6). Seventeen reference chromosomes were characterized by super-scaffolds representing either one full-length haplotype and one partial haplotype, two partial haplotypes, a single full-length haplotype or a single partial haplotype (25 super-scaffolds; Table S6, Fig. S6). Reference chromosome 5 was the only exception and was represented by one full-length haplotype and two partial haplotypes (Table S6, Fig. S6). Resolution of the remaining 11 reference chromosomes as distinct haplotypes was even less straightforward; two reference chromosomes (chr30 & chr31) were not confidently resolved while nine mapped to nine super-scaffolds in what can be described as ‘hybrid-hybrids’. These super-scaffolds corresponded to single Bionano-Canu scaffolds with each half of the scaffold representing a different reference chromosome (either a partial or full-length haplotype) or, in a single case, a Bionano-Canu scaffold containing portions of four different reference chromosomes (Figs. S7 & S8). No fewer than fourteen reference chromosomes were identified as contributing to portions of the hybrid-hybrids, with some reference chromosomes appearing more than once.

Perhaps not surprisingly, deeper investigation of the nine hybrid-hybrid super-scaffolds, their respective Canu-contig sequences, and homologous reference chromosomes revealed that LTR-RT insertions and segmental duplications were a confounding factor in their formation. In three cases (SS100002, SS00015, SS100022), Bionano appeared to erroneously resolve contigs to single super-scaffolds when segmental duplications (consistent with those detected for the *P. tricornutum* reference genome [11]) and/or LTR-RTs were present near the contig ends (Fig. S7). The similar labeling enzyme patterns of those repetitive genomic regions appear to have been detected by the Bionano software as portions of a single molecule that should be joined in a single molecule map. Interestingly, the resolution of hybrid-hybrid super-scaffold SS100022, which linked contigs homologous to reference chromosomes 24 and 29 (Fig. S7B), is consistent with Diner et al. [62]; these authors identified similar putative centromere sequences located at the termini of those scaffolds (which also lack telomeres) and hypothesized that chromosomes 24 and 29 are in fact two portions of a single chromosome. Our Bionano data support the genomic arrangement theorized by Diner et al. [62], but due to the repetitive nature of the area where the Canu contigs are joined, we could not confidently join chromosomes 24 and 29.

Assessment of the remaining eight hybrid-hybrid super-scaffolds was more complicated. For four of these hybrid-hybrids, we observed that the portions of the scaffold identified as coming from different chromosomes were separated by one or more large gap regions (Fig. S8A-D). In other cases, the ‘breakpoints’ between the inter-chromosomal mergers occurred in the middle of a single Canu contig (Fig. S8E-F). To assess if those Canu contigs represent mis-assemblies produced by the Canu assembly process, we mapped the raw nanopore long-read sequences to the Canu contigs in question. In both cases, we identified multiple long-reads spanning the ‘breakpoint’. Clearly there are artefacts being introduced but with the data in hand we cannot determine where and why.

To determine if some of the 155 unscaffolded contigs that did not meet the minimum size requirement for inclusion in the Bionano optical mapping could be used to manually complete partially resolved haplotypes and fill in gap regions inserted into the Bionano-Canu super-scaffolds, we aligned the Bionano super-scaffolds to their respective homologous reference chromosomes. Based on these alignments, we used blastn to compare the unscaffolded contigs against the specific reference chromosome regions determined to be missing from our partially resolved haplotypes. The creation of a more complete haplotype was straightforward in cases where a partial haplotype for a given reference chromosome was also represented by a full-length haplotype, which could be used as a guide for positioning the homologous unscaffolded contigs. However, in cases where a chromosome was represented by two partial haplotypes, correct haplotype assignment was not possible due to a lack of genomic context (File S1).

Given that our Bionano data support the separation of the Canu contigs into two haplotypes, we attempted to confirm that the original reference genome is indeed the product of haplotype amalgamation. A SNP frequency analysis was performed using only reference chromosomes for which two full-length haplotypes were represented (five case studies in total). First, long-read data were mapped to the Canu contigs representing the full-length haplotypes and their corresponding reference chromosome. The two Canu haplotypes were then aligned to the appropriate reference genome scaffold and manually examined to compare the sites of difference (Fig. S9). SNP visualization for all five chromosomes examined strongly suggests that the reference chromosomes are a mixture of the two haplotypes resolved by Canu and supported by Bionano optical mapping (Table S7).

To assess the possibility that it is the Canu contigs that are the amalgams and that the published reference chromosomes represent only one of the two haplotypes, Illumina short reads were mapped to the Canu haplotypes and reference chromosomes. The SNP differences across individual Illumina reads (~ 120 bp) were consistent with the differences observed across the long-read contigs/haplotigs, further validating the notion that it is the reference sequence of Bowler et al. [11] that is mosaic. Roughly an equivalent number of Illumina reads were found to support each haplotype, which is consistent with the diploid nature of *P. tricornutum* (Fig. S10)*.*

The level of allelic divergence in our *P. tricornutum* data was clearly significant enough for the Canu assembly algorithm to separate the two haplotypes rather than collapsing them together. We wanted to determine whether such high levels of allelic differences could influence the Bionano results. More specifically, could the underlying haplotype sequence differences give rise to heterogeneous Bionano enzyme labelling sites and thus compromise the ability of the Bionano approach to find the two haplotypes? The Bionano system converts images of electrophoretically separated, fluorescently labelled, long DNA molecules into virtual molecules, which are then clustered into virtual consensus maps [36]. These consensus maps are then compared to reference sequences that have been computationally labeled at sites with the same motif. This enables the pattern of fluorescently labelled enzyme sites in real DNA molecules (as represented by the Bionano consensus maps) to be compared to the equivalent sequences in a long-read assembly. This could potentially allow the assembly to be separated into haplotypes as the contigs are oriented and aligned into larger, chromosome-level scaffolds. The ability of the Bionano software to distinguish between haplotypes is dependent on the sites selected for incorporating the dye, as well as the density of the sites selected for labeling. If there are too few labeling sites in the DNA, there may not be enough information to make informative patterns to ascertain haplotypes. Conversely, if there are too many sites, the labeling pattern can become distorted and unreliable.

In *P. tricornutum,* the direct labeling enzyme DLE-1 (CTTAAG) was selected from a limited number of direct labeling enzymes. When we evaluated the DLE-1 enzyme sites and SNPs across the Canu haplotypes, we found that 15% of enzyme sites were altered by allelic differences, indicating that one of the haplotypes would have fewer enzyme sites available and, as a result, a lower labeling density. The labeling density across the whole *P. tricornutum* genome was 7.51 sites/100 Kbp, which falls just below Bionano’s recommended labeling density of 8–25 sites/100 Kbp [63]. The reduced labeling density resulting from the altered enzyme sites likely further compromised the resolution of the Bionano approach and resulted in an inaccurate dye pattern. The combination of low labeling density and high SNP diversity appears to have impacted the number of available enzyme labeling sites and contributed to the inability of Bionano to fully phase both *P. tricornutum* haplotypes. Additionally, the low labeling density likely contributed to the generation of the hybrid-hybrid Bionano-Canu contigs.

All things considered, while the Bionano data validate the separation of the Canu contigs into haplotypes, neither Bionano nor nanopore sequencing (together or in isolation) was able to fully phase the *P. tricornutum* genome.

### Repetitive DNA and long-terminal repeat retrotransposon content in diatom genomes

A prominent feature of the *P. tricornutum* and *T. pseudonana* genomes is the presence of repetitive elements. We first explored this using RepeatMasker to identify, characterize and compare repetitive content among the polished long-read assemblies and reference genomes for both organisms. Repetitive elements were found to contribute 11.3 Mbp (19.9%) and 2.55 Mbp (7.6%) to our de novo *P. tricornutum* and *T. pseudonana* genome assemblies, respectively (Tables S8 & S9). These proportions represent more than a two-fold increase in repeat content relative to our reassessments of the published reference genomes [*P. tricornutum* 8.1% (2.22 Mbp) and *T. pseudonana* 3.2% (1.03 Mbp); Tables S8 & S9]. Transposable elements (TEs) comprised ~ 41% more of the *T. pseudonana* Flye assembly (1484 TEs, 3.8%, 1.27 Mbp; Table S9) than the reference genome TE content reassessed using the same RepeatMasker parameters (1049 TEs, 1.4%, 0.45 Mbp; Table S9). We identified a 3.3-fold increase in the number of TEs found in our *P. tricornutum* Canu assembly (5605 TEs, 15.9%, 9.05 Mbp; Table S8) versus the reference genome (1706 TEs, 6.4%, 1.76 Mbp; Table S8). Consistent with the results of Rastogi et al. [14], we detected a small proportion of the *P. tricornutum* genome (0.2%, 0.13 Mbp) as being comprised of short interspersed nuclear elements (SINES; a type of non-long terminal repeat retrotransposon), which were undetected in the original reference genome annotation [11]. To date, SINES have only been reported in two other diatom species, *Cyclotella cryptica* [25] and *Skeletonema costatum* [23] and their genomic impact and functional roles are not well understood.

The most dramatic difference in TEs between the *T. pseudonana* Flye and reference assemblies and the *P. tricornutum* Canu and reference assemblies was in the number of full-length, decaying and nested *Ty1*/*copia*-like long terminal repeat retrotransposons (LTR-RTs). While the *T. pseudonana* Flye genome had a ~ 1.5-fold increase in *Ty1*/*copia*-like LTR-RTs when compared to the reference genome, an even more striking pattern was observed in *P. tricornutum.* Whereas *Ty1*/*copia*-like LTR-RTs comprise 5.7% of the *P. tricornutum* reference genome (1383 LTR-RTs, 1.57 Mbp; Table S8), they were classified as an even larger fraction of the Canu genome assembly at 14.4% (4703 LTR-RTs, 8.18 Mbp; Table S8).

Previously, diatom *Ty1*/*copia*-like LTR-RTs were classified into seven *copia-*like groups (called CoDi for *copia-*like in diatoms) with six CoDi groups forming two diatom-specific *copia* lineages [3, 11, 15]. Several studies have elucidated the role that *Ty1*/*copia*-like LTR-RTs have played in diatom diversity, genome structure and ecological adaptation [15–17]. While LTR-RTs have been identified in a number of other diatom genomes as well, e.g., those of *Fragilariopsis cylindrus*, *Pseudo-nitzschia multistriata*, *Pseudo-nitzschia multiseries* and *T. pseudonana*), these genomes lack the degree of CoDi expansion seen in *P. tricornutum*. As part of a larger genome reannotation study, Rastogi et al. [14] reassessed repetitive content in *P. tricornutum* and reported a greater proportion of CoDi elements (~ 7.6%) than earlier estimates (~ 5.4%) by Maumus et al. [15]. The genomic fraction of CoDi elements reported by Maumus et al. [15] and Rastogi et al. [14] included full-length, decaying and nested CoDi elements. To provide greater insight into the proportion of full-length, putatively active *Copia-*type and *Gypsy-*type LTR-RTs potentially contributing to *T. pseudonana* and *P. tricornutum* genome evolution*,* we assessed both genomes with rigorous, optimized software programs for de novo LTR-RT discovery. This involved consideration of the key signatures of LTR-RTs, namely gag-pol genes, LTR sequences at each end, and target site duplication sequences directly flanking each LTR.

We identified 22 full-length CoDi and Gypsy loci in the original *T. pseudonana* reference genome; this included 10 additional putatively active loci that were overlooked in previous analyses (Table S10). For the de novo *T. pseudonana* Flye genome, we detected a total of 38 putatively active LTR-RTs. These CoDi or Gypsy elements were characterized as “previously reported loci” (i.e., loci homologous to those previously reported by Maumus et al. [15] in the reference genome), “overlooked loci” (loci homologous to those present in the reference genome but not reported) and “novel loci” (i.e., loci detected in our long-read assembly but without a homologous insertion in the reference genome). Unexpectedly, we identified only eight previously reported loci, four overlooked loci, and 26 novel CoDi and Gypsy insertions in the Flye *T. pseudonana* genome assembly (Table S10, Fig. S11). Using LTR-retriever [64], the insertion time for 14 of the 26 novel insertions was estimated to be zero, consistent with the possibility that these 14 insertions represent very recent LTR-RT insertions absent from the original reference genome (i.e., present in our *T. pseudonana* culture but not in that used for the initial reference genome). That said, it is possible that these novel insertions were present in some but not all of the original reference genome sequence and did not make their way into the final consensus due to bioinformatic constraints associated with the handling of alleles.

**Fig. 3:**
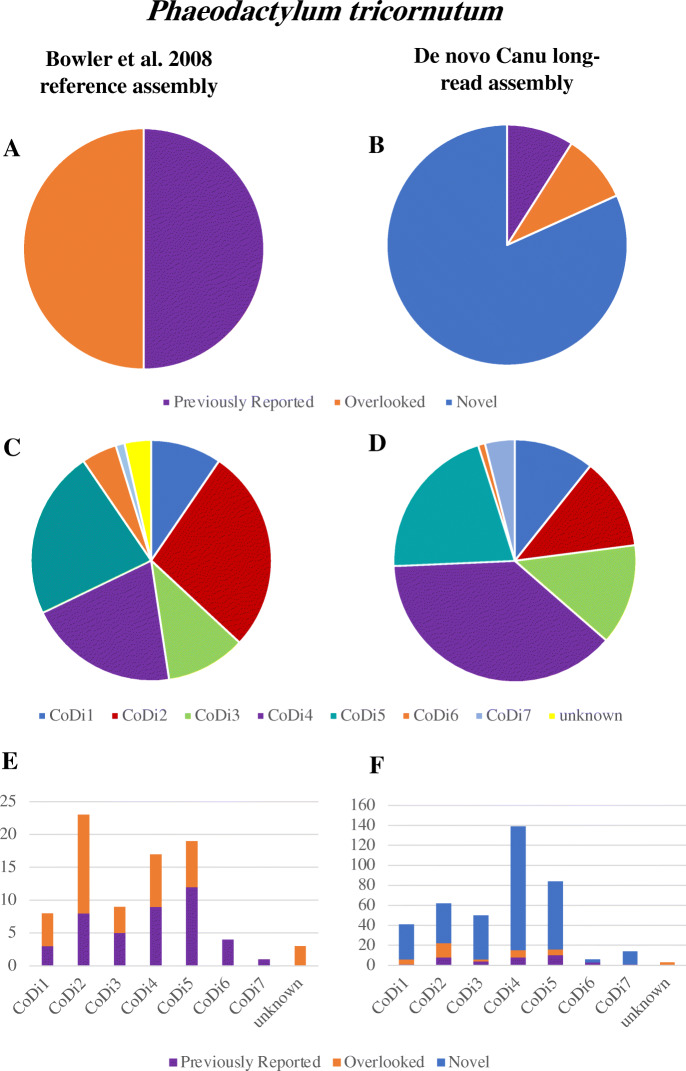
Full-length CoDi long-terminal repeat retrotransposon content in the *Phaeodactylum tricornutum* genome [11]*.* LTR-RT characterization is presented for the Bowler et al. reference genome (**a**) and Canu assembly (**b**). The Relative abundance of full-length LTR-RTs assigned to each CoDi group is presented for the Bowler et al. reference genome (**c**) and our de novo Canu assembly (**d**). Characterization of the LTR-RT loci resolved per CoDi group for the reference genome (**e**) and Canu assembly (**f**). LTR-RTs are characterized as either “previously reported loci” (i.e., loci homologous to those previously reported by Maumus et al. [14] in the reference genome), “overlooked loci” (those homologous to those present in the reference genome but not reported) or “novel loci” (i.e., loci detected in our long-read assembly but without a homologous insertion in the reference genome)

Results obtained for *P. tricornutum* were even more striking. Detection of LTR-RTs in the reference genome confirmed the 42 full-length previously reported CoDi elements and an equal number of overlooked full-length CoDi loci (Table S11, Fig. 3). We identified the CoDi5 group as a main contributor to the LTR-RT expansion, in addition to the previously recognized CoDi2 and Codi4 groups [15] (Table S11, Fig. 3)*.* Out of the 84 LTR-RT insertions detected in our search, 73 (87%) were identified in the *P. tricornutum* Canu assembly (36 previously reported loci and 37 overlooked loci, Table S11). In addition to those 73 loci, we detected 327 putative novel CoDi insertions in our Canu genome assembly (Table S12, Fig. 3). It is worth noting that further analysis of the Canu contigs representing alternative haplotypes indicated that most CoDi insertions were located in only a single haplotype, which is consistent with the observations of Maumus et al. [15].

In an attempt to determine if those novel loci were indeed the product of recent LTR-RT proliferation in our cell culture since the reference genome was published, or if these loci were present in the data of Bowler et al. [11] but not identified due to bioinformatic processing steps, we analyzed the raw sequencing reads generated for the original *P. tricornutum* genome project and mapped them to the Canu genome. These analyses indicated that the vast majority of the novel loci uncovered for *P. tricornutum* are actually supported by the raw Sanger sequencing reads, although a small proportion of insertions (~ 10%, 33 novel insertions) were not supported by raw reads and thus presumably represent authentic novel CoDi insertions present in one or both alleles.

Our LTR-RT investigation of *P. tricornutum* indicates that there are far more full-length CoDi elements present in the genome than previously recognized. Out of the 8.18 Mbp of LTR-RT sequences estimated by RepeatMasker for the *P. tricornutum* Canu genome assembly, ~ 32% (~ 2.69 Mbp) were identified as full-length CoDi elements predicted to have the required structural and enzymatic components needed for activation. The rate at which these loci are actively proliferating, and the biological significance of this proliferation, remains to be determined.

## Conclusions

Like most genomes sequenced in the 2000s, the *T. pseudonana* and *P. tricornutum* reference genomes were generated by paired-end Sanger sequencing of small, medium and large insert libraries followed by extensive manual finishing to resolve low-quality, ambiguous and gap regions (and in the case of *T. pseudonana,* optical restriction site mapping). The end result was two highly contiguous (< 200 scaffolds) genome assemblies that formed the foundation for numerous breakthroughs in diatom biology, ecology and evolution. Although both genomes are relatively small in size, they contain large-scale duplications and rearrangements and, in the case of *P. tricornutum,* LTR-RTs which contribute a significant portion of the genome [3, 11]. These structural complexities present challenges for Sanger and short-read next generation sequencing methods, raising the possibility that the existing reference genomes contain mis-assemblies, erroneously resolved repetitive regions, and/or large structural variations that were not accounted for by early sequencing technologies. Our re-sequencing of the genomes of *T. pseudonana* and *P. tricornutum* was in large part an effort to determine the extent to which long-read sequencing and current assembly algorithms can replicate — and improve on — the contiguity and quality of genomes that were sequenced using ‘early’ genome sequencing and assembly methods.

While our de novo long-read derived assemblies for *T. pseudonana* and *P. tricornutum* are lacking in some regards (e.g., continuity and chromosome-level scaffolds) compared to the original reference genomes, they largely validate the genome structure and organization presented in the existing reference genomes and provide additional sequence information lacking therein. By resolving the sequences of gap regions in the original reference genomes, linking previously separated scaffolds, and reconciling the placement of the majority of the reference genome’s unanchored contigs, we have shown that revisiting and resequencing highly contiguous genomes with chromosome-level scaffolds has value. Our long-read assemblies were just as complete in terms of gene content (as measured by BUSCO) and when our *T. pseudonana* assembly was combined with transcriptome data from NCBI, we discovered 1862 previously unreported genes. Our long-read assemblies also enabled fine-scale analysis of rRNA and LTR-RT content for both diatom species. More specifically, we were able to provide a more robust and comprehensive perspective of the number and locations of previously collapsed rRNA operons and LTR-RTs. These results highlight one of the major benefits of long-read sequencing, i.e., the ability to resolve repetitive content even if it comes at the expense of contiguity. Our data also validate previous findings that LTR-RT content is high in the *P. tricornutum* genome, and in fact demonstrate the existence of an even larger number of full-length, putatively functional LTR-RTs than previously believed.

Long-read sequencing has the potential to give rise to highly contiguous scaffolds representing all or most of an organism’s chromosomes (see e.g., the recent Nanopore sequencing of the model nematode *C. elegans* [43]). However, we have shown that even for relatively small nuclear genomes, genome complexity (e.g., the presence of transposable elements) can result in challenges that even the latest long-read assembly algorithms struggle to overcome. At first glance, long-read derived assemblies can appear ‘worse’ than Sanger-based ones. But in our efforts to obtain the ‘best’ genome assembly statistics, we must not lose sight of the fact that the genome biology that complicates the assembly process is part of a complete understanding of the organism. The question of what makes an assembly ‘better’ or ‘worse’ ultimately depends on the questions one wants to address with the data.

Our identification of over 300 full-length LTR-RTs in *P. tricornutum* (most of which were overlooked in the reference genome), as well as the resolution of alternative haplotypes, compromised our ability to match the existing reference genome in terms of contiguity alone. Based on the most common benchmarks for comparing genomes (e.g., number of contigs, read length N50), our Nanopore-derived *P. tricornutum* assembly is ‘worse’ than the Sanger-based reference. However, the *P. tricornutum* and *T. pseudonana* assemblies contain information that was not known prior to our study, information that should prove valuable in continued efforts to understand diatom genome biology and evolution. We view our long-read assemblies as additional genomic datasets that do not replace but complement and enhance the existing Sanger-based reference genomes – in isolation, neither provides a complete picture of the *P. tricornutum* or *T. pseudonana* genomes. These assemblies will no doubt be replaced in the near future as sequence reads become even longer, basecalling becomes more accurate, and assembly algorithms become smarter.

## Methods

### Diatom culture conditions

Axenic cultures of *Phaeodactylum tricornutum* Bohlin (strain CCMP632 Pt1, which is synonymous to the strain CCMP2561 used by Bowler et al. [11]; see De Martino et al. [65]) and *Thalassiosira pseudonana* (strain CCMP1335 [3]) were obtained from the Provasoli-Guillard National Center for Marine Algae and Microbiota, Bigelow Laboratory for Ocean Sciences, USA. The *P. tricornutum* culture was grown as replicates in f/2 medium made with artificial sea water (based on Kester el al [66]) enriched with f/2 vitamins, f/2 trace metal solution and supplemented with NaNO_3_ and NaH_2_PO_4_ H_2_O as described by Guillard [67]. All *P. tricornutum* replicates were maintained under approximately 75 μmol photons m^− 2^ s^− 1^ at room temperature (20–22 °C) in a 12-h photoperiod with continuous aeration on a horizontal shaker. Replicates of *T. pseudonana* were grown in L1 medium made with artificial sea water enriched with f/2 vitamin and L1 trace metal solution and supplemented with NaNO_3_, NaH_2_PO_4_ H_2_O and Na_s_SiO_3_ 9H_2_O [67]. The *T. pseudonana* replicates were grown at 18 °C in a 12-h photoperiod under approximately 75 μmol photons m^− 2^ s^− 1^ with continuous aeration on a horizontal shaker. Culture sterility was assessed monthly by inoculating 100 μl of cell culture in 1 ml of LB media made with artificial sea water [68] to determine any bacterial contamination.

### Diatom DNA extraction

One of the critical parameters required for generating and sequencing long molecules of DNA is the isolation of highly pure, unsheared/intact, high molecular weight (≥20 Kbp) genomic DNA. Numerous precautions were taken to extract high-quality DNA necessary for nanopore sequencing including cell culture isolation during early exponential phase, which greatly reduced the proportion of degraded DNA, handling DNA with wide-bore tips and employing gentle inversions versus vortexing. Cultures of *P. tricornutum* and *T. pseudonana* were grown to an approximate density of 1.4 × 10^8^ cells/ml and 4.5 × 10^6^ cells/ml, respectively. Diatom cells were harvested (approximately 9 days post-transfer) for high-molecular-weight genomic DNA extraction by centrifugation at 4 °C for 15 min at 2500×g. Cell pellets were resuspended in 1 mL of SDS lysis buffer (200 mM Tris-HCl pH 8, 250 mM NaCl, 25 mM EGTA, 0.5% w/v SDS) and subjected to 10 freeze/thaw cycles using liquid nitrogen and a 65 °C water bath. Proteinase K (20 mg/ml) was added (1 μl/100 μl of SDS lysis buffer) to the lysed cells and the samples were incubated at 50 °C for 60 min with gentle inversion every 15 min. RNase A (10 mg/ml) was added (5 μl /1 ml SDS lysis buffer) and samples were incubated at 37 °C for 30 min with gentle inversion every five minutes. Proteins were removed using two phenol:chloroform:isoamyl alcohol (25:24:1) extractions followed by two chloroform:isoamyl alcohol (24:1) extractions to remove any remaining phenol. Genomic DNA was precipitated with room temperature isopropanol and resuspended in pre-warmed 10 mM Tris-HCl, pH 8.0.

The integrity of the DNA was visually assessed using a 1% w/v agarose gel, aiming for a high-molecular-weight DNA band (> 20 Kbp) with little to no degradation. Quality of the DNA was determined by measuring A260/280 (goal ratio ~ 1.8) and A260/230 (goal ratio 2.0–2.2) using a NanoPhotometer P360 (Implen). The quantity of DNA was calculated using a Qubit 2.0 Fluorometer (ThermoFisher Scientific) and dsDNA broad range assay kit (ThermoFisher Scientific).

### MinION library preparation and sequencing

A DNA library was prepared for each diatom species using the Oxford Nanopore Technology (ONT) 1D Ligation Sequencing Kit (SQK-LSK108) and the “1D gDNA selecting for long reads” (version GLR1E_9022_v18_revT_18Oct2016) protocol with the following modifications. Pure (A260/280 = ~ 1.8, A260/230 = 2.0–2.2), unfragmented and non-size-selected *P. tricornutum* gDNA (6.7 μg) and *T. pseudonana* gDNA (5.5 μg) were used to prepare separate DNA libraries for two MinION R9.4 SpotON flow cells (FLO-MIN106). The DNA samples were repaired using the NEBNext FFPE DNA repair module (New England Biolabs cat. no. M6630) followed by a 0.45x AMPure XP bead (Beckman) clean-up in which 70 μl of resuspended beads were added to the 155 μl FFPE repair reaction and incubated at room temperature for 15 min, pelleted on a magnet and washed twice using 80% ethanol. The FFPE-repaired DNA was end-prepped using the NEBNext End repair/dA-tailing module (New England Biolabs cat. no. E7546) with extended incubation times of 30 min at 20 °C and 30 min at 65 °C. The end-prep step was followed by a 1x AMPure bead clean-up with the aforementioned modifications and extended incubation (15 min at 37 °C) of the resuspended bead pellet in nuclease-free water to improve long-molecule elution off the beads. The 1D adapter ligation was extended to 15 min at 25 °C and followed by a 0.6x AMPure bead clean-up with the previously mentioned extended incubation step and 80% ethanol washes. A total of 2 μg of prepared *P. tricornutum* library and 1.7 μg of prepared *T. pseudonana* library was loaded onto separate MinION R9.4 SpotON flow cells (FLO-MIN106), which were primed according to the specifications outlined by ONT. The “NC_48Hr_Sequencing_Run_FLO-MIN106_SQK-LSK108” sequencing script was run via MinKNOW software (v1.7.14) without live basecalling.

### MinION Bioinformatic processing

#### Raw read processing

The raw fast5 data generated by the MinION workflow were basecalled using Albacore (v2.1.7), which separates reads based on quality score (q-score) into “pass” (q-score > 7) and “fail” (q-score < 7) bins. The “pass” fastq files were processed with Porechop (v0.2.3) [69] to remove the 1D sequencing adapters. Subsets of the long-read datasets were created based on read length and read quality (−-mean-q-weight = 8) using the program Filtlong (v0.1.0) [70]. The filtered dataset for *P. tricornutum* included selection of the highest quality reads ≥20 Kbp for ~100x coverage of the expected genome size of ~28Mbp (−-target_bases = 2,700,000,000). The filtered *T. pseudonana* dataset included reads ≥30 Kbp for 100x coverage of the expected genome size of ~35Mbp (−-target_bases = 3,500,000,000). All read statistics and data plots for the unfiltered and filtered datasets were generated using NanoPlot (v0.20.1) [71].

#### Genome assembly

The filtered reads were assembled using the dedicated long-read assemblers, Canu (v1.6) [49] and Flye (v2.3) [51]. The complete Canu assembly pipeline (read correction, read trimming and unitig construction) was run with the following modifications made to the default parameters: correctedErrorRate = 0.13 and cnsErrorRate = 0.25. The Flye assemblies were generated with the default settings except for the minimum overlap between reads (−-min-overlap), which was set to 5 Kbp, and the number of polishing iterations (−-iterations), which was set to three.

#### Genome assembly correction

The Canu and Flye assemblies were corrected using a combination of MinION long-reads and Illumina short-reads (see below). First, the assembly was corrected by long-read data using two rounds of Racon (v1.3.1) [52] according to the default settings. The overlap information required by Racon was generated using Minimap2 (v2.5-r572) [72] with the “map-ont” preset option that is designed for use with Oxford Nanopore data. The Racon-corrected assemblies were further polished with the default Nanopolish (v0.10.1) [73] pipeline, which extracts signal-level data from the raw fast5 files to generate an improved polished consensus sequence. Finally, MinION long-read data and trimmed, PCR-free, paired-end Illumina short-read data (see below) were used to polish the assemblies using Unicycler-polish (v0.4.4) [74], which implements an iterative polishing program, Pilon (v1.22), [33]. Default parameters were used for both Unicycler_polish and Pilon. Pilon was run for at least ten iterations for each assembly.

#### Genome assembly evaluation & comparison

Genome assembly statistics were generated using the QUAST web interface [75]. The software Assembly Likelihood Evaluation (ALE) [54] was used to generate an overall assembly likelihood score for each assembly (including all polished variations). The ALE score was used to compare different assemblies of the same genomic sequence data to assess assembly accuracy. To run ALE, the program Bowtie2 (v2.3.1) [76] was used to map short-read data to each assembly. The resulting bam-file was provided to the ALE software to generate statistical values for comparison. Genome completeness of each assembly (including polished iterations) was assessed using the BUSCO (v3.0.2) [55] Eukaryota_odb9 database. The ploidy level was determined for the final polished working assemblies for each diatom using the ploidy assessment and visualization program ploidyNGS (v3.0) [77] with default parameters. The input bam-file used by ploidyNGS was generated by mapping Illumina reads to the draft assemblies using Bowtie2. Repetitive content in the final polished working assemblies was identified, classified and masked using RepeatMasker (v4.0.7) [78] with NCBI/RMBLAST 2.6.0+.

Mapping of long-read data to the long-read de novo assemblies and existing reference genomes was performed using the long-read mapper NGMLR (v0.2.7) [79], which was designed for effective read mapping despite structural variations. The program was run using the default settings and the parameter: “-x ont”. Short-read Illumina data were mapped to the draft and existing reference genomes using Bowtie2. Average read depth coverage was calculated using an in-house perl script.

The final polished Canu and Flye assemblies were compared to the existing reference genomes (*P. tricornutum*, https://genome.jgi.doe.gov/portal/Phatr2/Phatr2.download.html [11]; *T. pseudonana*, https://genome.jgi.doe.gov/portal/Thaps3/Thaps3.download.html [3, 11] by aligning the draft assemblies against the reference using NUCmer (v3.1, MUMmer package v3.0) [80]. The resulting delta alignment file was assessed by the web interface program Assemblytics [56] to report structural variation between the draft and reference genomes. The parameters for Assemblytics were set to the following: unique sequence length required = 1000, maximum variant size = 50,000, minimum variant size = 25.

The polished Canu and Flye assemblies were also compared to the existing reference genomes using the program Mauve [81] via the Mauve plug-in for Geneious (v11.1.5) [82] The Mauve Contig Mover (MCM) alignment algorithm was used to reorder and align the draft contigs relative to the original reference genome. The draft-to-reference alignments were visualized in Geneious and manually analyzed to assess large-scale genome rearrangements.

#### Chromosomal resolution of previously unplaced sequence data

All sequences (“bottom drawer” sequences) that could not be assigned to chromosomes or organelles from both diatom reference genome sequencing projects were downloaded from NCBI and compared against the appropriate diatom species long-read assembly using blastn (−evalue 1e-10 -qcov_hsp_perc 90). Placed contigs were validated manually using local alignment tools in Geneious.

#### Resolution of gaps in existing reference genomes

Gaps inserted in the original reference genomes for each diatom were assessed by manually inspecting MAFFT local alignments of the reference chromosomes and their homologous long-read derived contigs. Gaps were determined as resolved if a long-read contig spanned the inserted gap as well as the nucleotide sequence flanking the start and stop coordinates of the gap.

#### Assessment of complete rRNA repeats

To assess whether the final polished working assemblies for both diatoms included complete ribosomal operons (18S, ITS1, 5.8S, ITS2, 28S), relevant representative diatom rRNA sequences (full and partial rRNA) were downloaded from GenBank and used to screen the long-read assemblies using blastn. De novo contigs containing rRNA sequence were manually inspected and annotated using Geneious. Copies of rRNA for each diatom were aligned using the MAFFT (v7.450) [83] plug-in for Geneious and percent identity was calculated based on those alignments, also in Geneious. The average Illumina read depth coverage for rRNA loci was calculated using an in-house perl script.

### Illumina library preparation and sequencing

High molecular weight gDNA for both diatoms was provided to Genome Quebec (http://www.genomequebec.com/en/home/; Quebec, Canada) for the construction of a PCR-free, 2 × 150 bp paired-end library that was sequenced on the Illumina HiSeqX platform.

### Illumina Bioinformatic processing

Short-read data quality was assessed using FastQC (v.0.11.5) [84]. Sequencing adapters were removed using Trimmomatic (v0.36) [85], which was also used for filtering the reads according to the following parameters: -phred33, HEADCROP:20, LEADING:10, TRAILING:10, SLIDINGWINDOW:10:25, MINLEN:40.

### Gene prediction

The protein sequence dataset (PRJNA34119) associated with the original reference genome for *T. pseudonana* was downloaded from NCBI and compared to the Flye de novo assembly using tblastn.

Four sets of paired Illumina RNA-Seq SRA datasets (SRR9042946, SRR9042947, SRR9042958, SRR9042959) for *T. pseudonana* CCMP1335 were downloaded from NCBI. The reads were filtered for quality and length using Trimmomatic, mapped against the Flye de novo assembly with hisat2 (v2.2.0) [86] and assembled with Trinity (v2.9.1) [87]. The Trinity assembled transcriptome was compared against the NCBI reference nucleotide dataset (Accession GCF_000149405.2_ASM14940v2_rna.fna) of protein coding genes using blastn (evalue 1e-15). The Trinity assembled transcriptome was then compared against the NCBI nucleotide database using blastn (evalue 1e-15). The Trinity assembled transcriptome was also compared against the original reference genome and the Flye de novo assembly.

The Flye de novo assembly and Trinity transcriptome were used for gene prediction using an in-house pipeline based on BRAKER (v2) [88] with increased attention to chimeric gene models and real intron boundaries. The gene set was then corrected using PASA [89]. The resulting protein coding gene dataset was compared to (1) the reference protein coding gene dataset using blastp and (2) the NCBI protein database using diamond blastp.

Using PLAST [90], the PASA predicted gene dataset and Trinity transcriptome predicted for the Flye de novo assembly were compared against published gene datasets for *Phaeodactylum tricornutum* [11, 14], other diatom gene datasets from NCBI as well as gene datasets for other stramenopiles downloaded from NCBI.

Broccoli (v1.2) [91] was used to infer orthologous groups from the complete predicted protein datasets for *T. pseudonana* and seven other diatom species (*Fistulifera solaris, Fragilaria radians, Fragilariopsis cylindrus, Phaeodactylum tricornutum, Pseudo-nitzschia multiseries, Pseudo-nitzschia multistriata* and *Thalassiosira oceanica*). The proteomes for seven outgroup taxa (*Arabidopsis thaliana, Bigelowiella natans, Dictyostelium discoideum, Guillardia theta, Homo sapiens, Trypanosoma brucei, Saccharomyces cerevisiae*) were downloaded from NCBI and included to increase precision and accuracy. The following settings were specified for the second step of Broccoli, which uses DIAMOND (v0.9.25+) [92] and FastTree (v2.1) [93] to perform similarity searches for each query protein, build pairwise alignments and then phylogenetic analyses: “-e_value 0.001 -nb_hits 6 -phylogenies ml”. Output files generated by Broccoli were parsed using in-house python scripts. Further assessment of the protein sequences assigned to orthologous groups by Broccoli was performed using PLAST (v2.3.2) [90].

### Bionano optical mapping

Live *P. tricornutum* culture was provided to HistoGenetics (https://www.histogenetics.com/; NY, USA) who prepared genomic DNA using in-house protocols. The polished Canu long-read assembly was provided to HistoGenetics and analyzed to identify potential labelling enzymes based on their respective recognition sites. The prepared DNA was then labelled using a Direct Label and Stain reaction with enzyme DLE1 (recognition site: CTTAAG). Post-labeling, whole genome optical mapping was performed by HistoGenetics using the BioNano Saphyr platform and the long-read contigs from the Canu assembly were scaffolded with the Bionano maps. Obtained data were analyzed using Bionano Genomics graphical interface, Geneious and in-house scripts.

The hybrid Canu-Bionano scaffolds were localized to their homologous reference chromosomes using blastn. Sequence data was resolved for the gaps inserted in the Bionano scaffolds by (i) using Mauve to align each Bionano scaffold to its homologous reference chromosome from Bowler et al. (2008), (ii) manually inspecting the Mauve alignment to identify regions of reference chromosome sequence data that resolve in the Bionano scaffold gap regions, and, (iii) using blastn to query the “missing reference chromosome regions” from step 2 against a local database of contigs (< 150 Kbp) excluded from the Hybrid assembly.

SNP frequencies for the *P. tricornutum* Canu contigs were determined by mapping 17,472,834 Illumina reads to the Canu assembly with histat2 (v2.2.0) [86] (−-score-min L,0,-0.6), finding SNPs with samtools mpileup [94] and then calling legitimate SNPs with an in-house perl script.

### Pulsed-field gel electrophoresis

Agarose plugs were prepared from *Phaeodactylum tricornutum* cell culture following the “Preparation of Agarose Embedded Mammalian DNA” protocol in the BioRad CHEF-DR III manual (http://www.bio-rad.com/webroot/web/pdf/lsr/literature/M1703690.pdf). The plugs were stored at 4 °C in 1x Wash Buffer (20 mM Tris, pH 8.0, 50 mM EDTA) and loaded into a 1% agarose gel (prepared using BioRad pulsed field certified agarose). Pulsed-field gel electrophoresis (PFGE) was run using the CHEF-DR III system according to the following conditions: resolution of short DNA fragments-- 0.5x TBE running buffer, 14 °C, 60 s initial switch time, 120 s final switch time, 42 h, 120° angle, voltage gradient of 4.5 V/cm; resolution of longer DNA molecules-- 1x TAE running buffer, 14 °C, 500 s switch time, 48 h, 106° angle, voltage gradient of 3 V/cm. After the PFGE run was completed, the gel was stained for 30 min in a 1 μg/ml Ethidium Bromide solution and imaged.

### Long-terminal repeat retrotransposon assessment

Full-length long-terminal repeat retrotransposons (LTR-RTs) for both diatom genomes were identified using three analyses. The tool LTR_finder (v1.07) [44] was used according to the following settings: -D 15000 -d 1000 -L 5000 -l 100 -p 20 -M 0.00 -w 2. Protein domains were predicted by invoking the “-a” setting to call on “ps_scan” based on the PROSITE database of protein families [95]. LTR-RT candidates from LTR_finder were further refined by screening the output file with the software LTR_retriever (v2.7) [64]. All diatom reference LTR-RTs from Maumus et al. [14] were downloaded from GenBank and compared to the appropriate diatom species de novo long-read genome assembly. The raw sequencing reads generated by Bowler et al. [11] were obtained from the Joint Genome Institute and aligned to the Canu assembly using Bowtie2. An in-house perl script was used to determine if any original reads spanned the boundaries of the LTR-RTs in the Canu genome.

## Supplementary Information

**Additional file 1: Supplementary File 1.** Case studies assessing if the 155 contigs that were too small for inclusion in the *Phaeodactylum tricornutum* Bionano-Canu hybrid assembly could be used to manually complete partially resolved haplotypes and close gap regions inserted into the Bionano-Canu super-scaffolds.

**Additional file 2: Supplementary Table 1.** Detailed raw read data summary for unfiltered, Albacore “pass” and filtered Oxford Nanopore long-read sequencing datasets for *Thalassiosira pseudonana* and *Phaeodactylum tricornutum*. The filtered datasets for *T. pseudonana* and *P. tricornutum* included reads ≥30 kb and ≥ 20 kb, respectively.

**Additional file 3: Supplementary Table 2.** Assembly statistics for various polishing iterations of the de novo long-read derived genomes for *Thalassiosira pseudonana* and *Phaeodactylum tricornutum*. Asterisks indicate the final working assembly for each species.

**Additional file 4: Supplementary Table 3.** Chromosome assignment of “Bottom Drawer” contigs for *Phaeodactylum tricornutum* and *Thalassiosira pseudonana.*

**Additional file 5: Supplementary Table 4.** Long-read contigs help close gaps in the *Phaeodactylum tricornutum* and *Thalassiosira pseudonana* reference genomes*.*

**Additional file 6: Supplementary Table 5.** Structural variation between the polished de novo Canu and Flye assemblies and the reference genomes for *Phaeodactylum tricornutum* and *Thalassiosira pseudonana*.

**Additional file 7: Supplementary Table 6.** Bionano hybrid assembly statistics report for *P. tricornutum* including haplotype assignment details.

**Additional file 8: Supplementary Table 7.** SNP frequencies for each contig in the *P. tricornutum* Canu genome.

**Additional file 9: Supplementary Table 8.** Comparison of RepeatMasker determined repetitive content for the final polished long-read working assembly and the reference genome for *Phaeodactylum tricornutum*. Note that the numbers reported for LTR elements include full-length, nested and decaying elements.

**Additional file 10: Supplementary Table 9.** Comparison of RepeatMasker determined repetitive content for the final polished long-read working assembly and the reference genome for *Thalassiosira pseudonana*. Note that the numbers reported for LTR elements include full-length, nested and decaying elements.

**Additional file 11: Supplementary Table 10.** Details of full-length, putatively active LTR-RT discovery for the *T. pseudonana* reference and de novo Flye genomes.

**Additional file 12: Supplementary Table 11.** Details of full-length, putatively active LTR-RT discovery for the *P. tricornutum* reference genome.

**Additional file 13: Supplementary Table 12.** Details of full-length, putatively active LTR-RT discovery for the *P. tricornutum* de novo Canu genome.

**Additional file 14: Supplementary Figure 1.** Workflow of sample preparation, MinION sequencing, de novo genome assembly and downstream analyses, including methods to compare the de novo long-read reference assemblies and gene prediction. **Supplementary Figure 2**. Bivariate scatterplots showing the relationship of MinION read lengths and average Phred read quality scores for *Phaeodactylum tricornutum* (A & B) and *Thalassiosira pseudonana* (C & D). The unfiltered datasets (A & C) include all generated MinION data and the filtered datasets (B & D) include a subset of reads filtered by length and quality. **Supplementary Figure 3.** Pulsed-field gel electrophoresis of *P. tricornutum* (CCMP632) DNA using settings to optimize resolution of large (A) and small (B) fragments. The red arrowheads (<) indicate potential chromosome-sized fragments. Ladders for sizing fragments include *Saccharomyces cerevisiae* (Sc) and *Hansenula wingei* (Hw). Lanes labelled 1–4 and 8–13 are not relevant to this study. **Supplementary Figure 4.** Relationship between 16,491 predicted protein models from the Flye *Thalassiosira pseudonana* assembly to reference protein set and the matches to known proteins of the new predicted protein models (2010). **Supplementary Figure 5**. PloidyNGS plot of the frequency of the two most abundant alleles in the *Phaeodactylum tricornutum* genome indicates that it is a diploid organism. **Supplementary Figure 6.** Stacked histograms showing the different categories of *P. tricornutum* haplotypes supported by Bionano hybrid scaffolding. Characterizations are based on a total of 66 haplotypes expected based on a diploid genome with 33 chromosomes as estimated for the reference genome. **Supplementary Figure 7.** ‘Hybrid-hybrid’ Bionano-Canu super-scaffolds that may represent mis-assemblies owing to segmental duplications (A & B) or LTR-RT insertions (C) located at the ends of Canu contigs. Mauve [81] schematics of the syntenic regions between a Bionano-Canu super-scaffold and the reference chromosomes that it contains demonstrate the hybrid nature of each super-scaffold. Inserts provide a more detailed illustration of the high sequence identity between the hybrid-hybrid scaffolds and the reference chromosomes at the areas of the genome containing segmental duplications or LTR-RT insertions. **Supplementary Figure 8.** Bionano-Canu super-scaffolds that were identified as ‘hybrid-hybrid’ scaffolds most probably owing to errors of the Bionano mapping process. For each example (A-F), a schematic of the super-scaffold is annotated with its respective Canu contigs shown in purple blocks. Gap regions inserted by Bionano are indicated by solid black lines. Syntenic regions between each ‘hybrid-hybrid’ super-scaffold and the two reference chromosomes it contains were evaluated by Mauve [81] and shown as colored blocks above the super-scaffold schematic. Blastn results are reported for each of the Canu contigs resolved to the ‘hybrid-hybrid’ super-scaffold against the appropriate reference genome chromosome. **Supplementary Figure 9.** Multiple sequence alignment showing a region of the *P. tricornutum* Canu assembly that is represented by two contigs (haplotype 1 = tig94 & haplotype 2 = tig92) while the reference genome is only represented by a single scaffold (chr3). The blue and red boxes indicate SNPs between the reference scaffold and Canu haplotigs. While the reference scaffold and haplotype 1 match at the first three SNPs, the reference scaffold disagrees with haplotype 1 at the following few SNPs, matching haplotype 2, instead. That pattern is strongly suggestive that the reference is an amalgamation of the two haplotypes resolved by the Canu assembly. Asterisks represent mapped Illumina short-reads with green boxes representing areas where individual ~ 120 bp Illumina reads supported the SNPs captured for each Canu haplotype. **Supplementary Figure 10.** IGV schematic showing the location of four SNPs between the reference genome and two Canu haplotigs. The SNPs are indicated by the four bi-colored columns, which correspond to the number of alternative bases detected at each site. The four SNPs are consistent across the mapped reads with the blue boxes representing haplotype 1 and the red boxes representing haplotype 2. Roughly equivalent numbers of reads were found to support each haplotype, which is consistent with *P. tricornutum* as a diploid genome. **Supplementary Figure 11.** Full-length CoDi long-terminal repeat retrotransposon content resolved for *T. pseudonana.* The number of previously reported & overlooked loci are reported for the reference genome (A) as well as the Flye assembly (B), which also included novel LTR insertions. The number of LTR-RTs detected for each CoDi group in the reference genome is compared to the number of LTR-RTs detected for each CoDi group in the Flye de novo assembly (C). LTR-RTs are characterized as either “previously reported loci” (i.e., loci homologous to those previously reported by Maumus et al. [15] in the reference genome), “overlooked loci” (those homologous to those present in the reference genome but not reported) or “novel loci” (i.e., loci detected in our long-read assembly but without a homologous insertion in the reference genome).

## Data Availability

The datasets generated and analyzed during the current study are available as follows: The raw fast5 MinION data have been deposited in the NCBI SRA database (*P. tricornutum* accession: SRX4617960; *T. pseudonana* accession: SRX4617979). Illumina sequence data have been deposited in the NCBI SRA database (*P. tricornutum* accession: SRX4617959; *T. pseudonana* accession: SRX4617978). The Bionano hybrid scaffold data have been deposited in NCBI (*P. tricornutum* accession: BioProject PRJNA487263, Supplementary Data accession SUPPF_0000003857). The polished Canu long-read assembly for *P. tricornutum*, polished Flye long-read assembly for *T. pseudonana* and protein-coding gene dataset for *T. pseudonana* are available at 10.5683/SP2/ZDZQFE. All other genomic assemblies are available upon request.

## Abbreviations

Mya: Million years ago
Mbp: Megabase pairs
EST: Expressed sequence tag
HGT: Horizontal gene transfer
TE: Transposable elements
LTR-RTs: Long terminal repeat retrotransposons
ONT: Oxford Nanopore Technologies
bp: Base pairs
Kbp: Ailobase pairs
Gbp: Gigabase pairs
Q-score: Quality score
ALE: Assembly Likelihood Evaluation
PFGE: Pulsed-field gel electrophoresis
rRNA: Ribosomal RNA operon
