# Supplementary material for: Re-examination of two diatom reference genomes using long-read sequencing
Source: BMC Genomics. 2021 May 24;22:379. doi: 10.1186/s12864-021-07666-3 (PMC8147415; doi:10.1186/s12864-021-07666-3)
Supplement: Supplementary file 1 — Additional file 1: Supplementary File 1. Case studies assessing if the 155 contigs that were too small for inclusion in the Phaeodactylum tricornutum Bionano-Canu hybrid assembly could be used to manually complete partially resolved haplotypes and close gap regions inserted into the Bionano-Canu super-scaffolds. [file 12864_2021_7666_MOESM1_ESM.pdf]

Supplementary Information for:

Re-examination of two diatom reference genomes using long-read sequencing

Gina V. Filloramo[1,2*], Bruce A. Curtis[1,2], Emma Blanche[1,2] & John M. Archibald[1,2*]

[1] Department of Biochemistry and Molecular Biology, Dalhousie University, Sir Charles Tupper Medical Building, 5850 College Street, PO Box 15000, Halifax, Nova Scotia, Canada B3H 4R2

[2] Centre for Comparative Genomics and Evolutionary Bioinformatics, Dalhousie University, Halifax, Nova Scotia, Canada

[*] To whom correspondence should be addressed: john.archibald@dal.ca; gina.filloramo@dal.ca

Supplementary File 1. Case studies assessing if the 155 contigs that were too small for inclusion in the *Phaeodactylum tricornutum* Bionano-Canu hybrid assembly could be used to manually complete partially resolved haplotypes and close gap regions inserted into the Bionano-Canu super-scaffolds.

BOWLER ET AL 2008 reference CHR3 (1,460,046 bp)

HAPLOTYPE 1: Super-Scaffold_100010 → telomere to telomere

- 1,772,416 bp (length supported by PFGE)

1	408336	Consensus_Consensus_tig00002792_subseq_896526:1304861 (-)
408337	408349	GAP 13
408350	1772416	tig00000092 (+)

- Includes 2 canu contigs

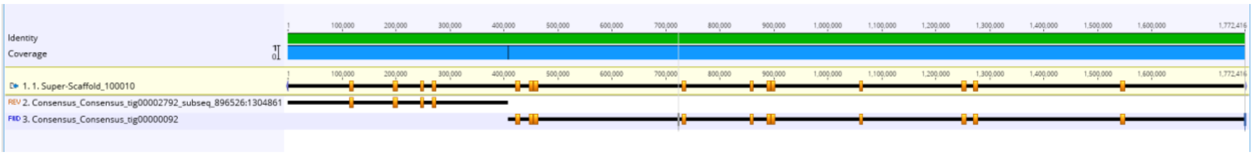

HAPLOTYPE 2: Super-Scaffold_100012 → no telomeres resolved

- 1,012,550 bp
- Includes 3 canu contigs

1	158602	Consensus_Consensus_tig00000102 (-)
158603	203941	GAP 45,339[1]
203942	290375	Consensus_Consensus_tig00000097 (+)
290376	904404	GAP 614,029[2]
904405	1012550	Consensus_Consensus_tig00000096 (+)

***Note that SS_100012 is syntenic to the following regions of BOWLER ET AL 2008 reference chromosome 3: ~382711-531073; 571269-651974; 1220821-1322320. So SS_100012 is lacking homology to chr3: 1-380 kb and chr3: 137-1400 kb. A number of unscaffolded contigs were identified with blastn as homologous to reference chr3 to fill in the missing portions at the start and end of SS_100012.

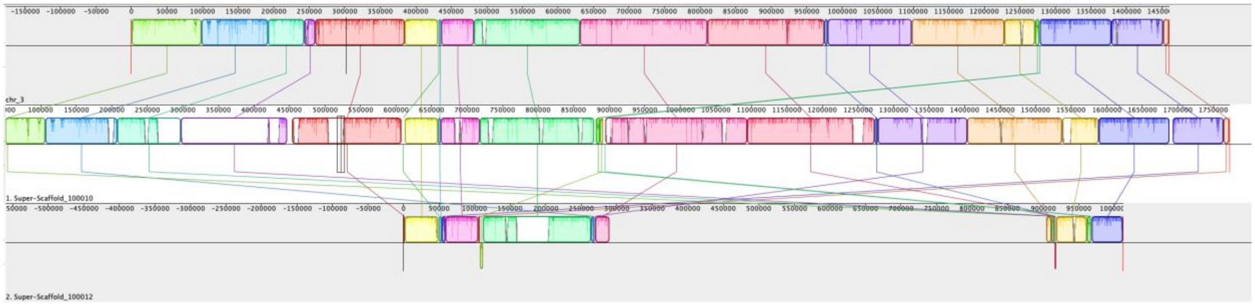

<u>The following five unscaffolded contigs extend the 5P end of SS_100012</u>

Consensus_tig00000105 (91,048 bp) overlaps with the 5P end of SS100012 and extends the length of the scaffold 66,879 bp

S1	S1 start	S1 stop	S2	S2 start	S2 stop
tig00000105	24169	2	SS_100012	2	24154

S1	S1 start	S1 stop	S2	S2 start	S2 stop
tig00000105	91046	3	chr3	328502	406926

Consensus_tig00000103 (60,096 bp)

S1	S1 start	S1 stop	S2	S2 start	S2 stop
tig00000103	41766	60093	tig00000106	103430	85080

S1	S1 start	S1 stop	S2	S2 start	S2 stop
tig00000103	1	60096	chr3	326384	266242

Consensus_tig00000106 (103,432 bp)

S1	S1 start	S1 stop	S2	S2 start	S2 stop
tig00000106	1	103432	chr3	188337	284603

S1	S1 start	S1 stop	S2	S2 start	S2 stop
tig00000106	103430	85080	tig00000103	41766	60093

Consensus_tig00000100 (168,241 bp)

S1	S1 start	S1 stop	S2	S2 start	S2 stop
tig00000100	31693	168241	chr3	10366	131085

Consensus_tig00002793 (69,984 bp)

S1	S1 start	S1 stop	S2	S2 start	S2 stop
tig00002793	1	67085	chr3	63251	1084

The following single unscaffolded contig extends the 3P end of SS100012

Consensus_tig00000111(121,339bp)

S1	S1 start	S1 stop	S2	S2 start	S2 stop
tig00000111	1	119556	chr3	1351081	1457872

[1] GAP is not resolved by unscaffolded contigs.

[2] GAP is resolved by Consensus_tig00000094, Consensus_tig0000099, Consensus_tig00000143.

Consensus_tig00000094 (70,965 bp) overlaps with the portion of SS_100012 that is located immediately upstream from the 5P end of the gap.

S1	S1 start	S1 stop	S2	S2 start	S2 stop
tig0000094	70963	65622	SS_100012	285029	290375

S1	S1 start	S1 stop	S2	S2 start	S2 stop
tig0000094	3	70963	Chr3	713389	646626

Consensus_tig00000099 (70,965 bp) note that this contig does not overlap with other contig assigned to SS_100012. The entirety of this contig aligns to chr3:1009204-930421. Its position relative to the gap region of SS_100012 is somewhat ambiguous as I cannot anchor it to any sequence data in SS_100012.

S1	S1 start	S1 stop	S2	S2 start	S2 stop
tig0000099	4	29945	Chr3	1009204	979888
tig0000099	34137	36167	Chr3	979883	969995
tig0000099	43666	83241	Chr3	969990	930421

Consensus_tig00000143 (83,162 bp) note that this contig does not overlap with other contig assigned to SS_100012. The entirety of this contig aligns to chr3: 1013462-1090876. Its position relative to the gap region of SS_100012 is somewhat ambiguous as it cannot be anchored to any sequence data in SS_100012.

S1	S1 start	S1 stop	S2	S2 start	S2 stop
tig0000143	6441	28105	Chr3	1090876	1068940
tig0000143	30515	47431	Chr3	1068937	1052035
tig0000143	48779	83160	Chr3	1052031	1013462

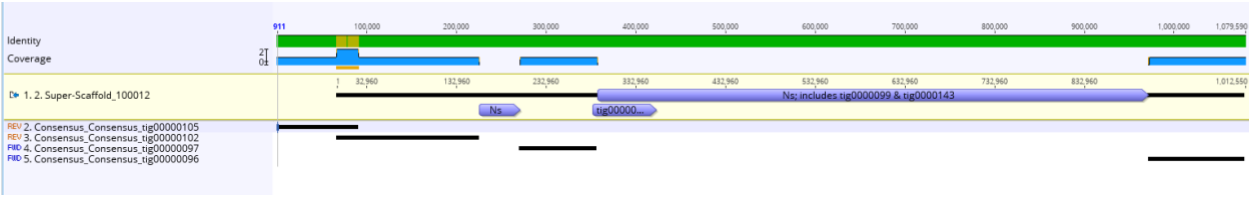

BOWLER ET AL 2008 reference CHR8 (1,007,773 bp)

HAPLOTYPE 1: Super-Scaffold_100021 → telomere to telomere

- 1,204,650 bp (length supported by PFGE)

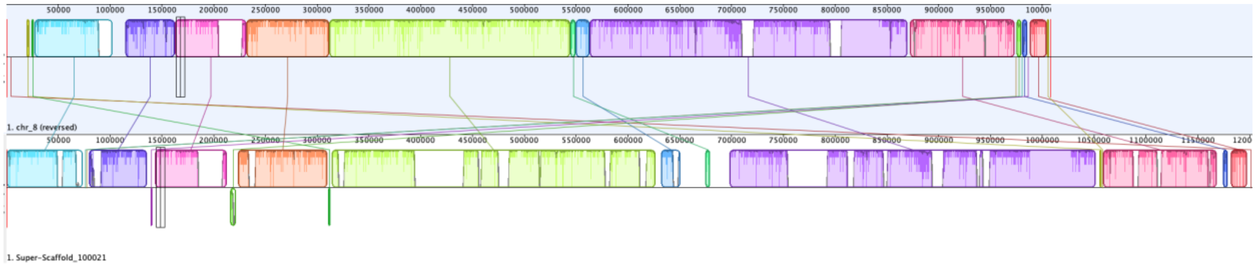

- Includes 6 canu contigs

1 184598 Consensus_Consensus_tig00000176 (-)

184599	208252	GAP insertion 23,654 bp[1]
208253	436163	Consensus_Consensus_tig00000363 (+)
436164	436176	GAP 13
436177	674551	Consensus_Consensus_tig00000364 (+)
674552	674564	GAP 13
674565	771567	Consensus_Consensus_tig00000365 (+)
771568	771580	GAP 13
771581	892961	Consensus_Consensus_tig00000384 (-)
892962	904090	GAP insertion 11,129 bp[2]
904091	1204650	Consensus_Consensus_tig00000218 (+)

[1] GAP is resolved with Consensus_tig00000179
- length: 69,770 bp
- coordinates:

S1	S1 start	S1 stop	S2	S2 start	S2 stop
SS100021	259134	208254	tig00000179	3	33521
SS100021 GAP	208252	184599	tig00000179	33522	56757
SS100021	184582	171550	tig00000179	56758	69768

- Consensus_tig00000179 blasts to BOWLER ET AL 2008 reference chr8

S1	S1 start	S1 stop	S2	S2 start	S2 stop
tig00000179	3	69768	Chr8	260722	190952

[2]GAP is not resolved with any of the unscaffolded <150 kb contigs

HAPLOTYPE 2: Super-Scaffold_100017 → no telomeres resolved*
- 1,228,605 bp (length supported by PFGE)

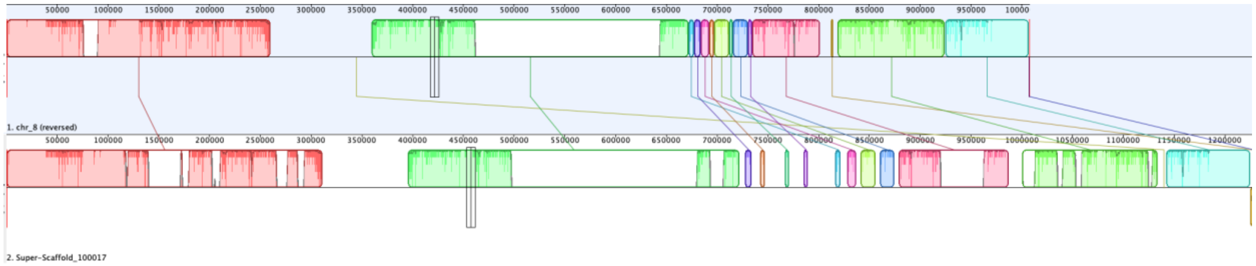

-*note that this contig is longer than HAPLOTYPE 1 which has both telomeres. Is this a result of gaps? Large insertions?

- Includes 6 canu contigs

1	159605	Consensus_Consensus_tig00000361 (+)
159606	159618	13
159619	311613	Consensus_Consensus_tig00000362 (+)

311614	395706	GAP 84,093 bp[1]
395707	496754	Consensus_Consensus_tig00000182 (-)
496755	679871	GAP 183,117 bp[2]
679872	814957	Consensus_Consensus_tig00000199 (-)
814958	814970	13
814971	938115	Consensus_Consensus_tig00000383 (-)
938116	938128	13
938129	1228605	Consensus_Consensus_tig00000382 (-)

[1] GAP is not resolved with any of the unscaffolded <150 kb contigs
[2] GAP resolved by Consensus_tig00000185 & Consensus_tig00000366

Consensus_tig00000185
- length: 148,468 bp
- coordinates:

S1	S1 start	S1 stop	S2	S2 start	S2 stop
SS100017	480624	496762	tig00000185	3	16145

note that the remaining ~120 kb of tig00000185 extends into the gap region

- Consensus_Consensus_tig00000185 blasts to BOWLER ET AL 2008 reference chr8

S1	S1 start	S1 stop	S2	S2 start	S2 stop
tig00000185	3	148466	Chr8	444928	591667

Consensus_tig00000366
- length: 64,458 bp
- coordinates:

S1	S1 start	S1 stop	S2	S2 start	S2 stop
SS100017	679918	706582	tig00000366	37818	64456

note that the first ~37 kb of tig00000366 is in the gap region

- Consensus_Consensus_tig00000366 blasts to BOWLER ET AL 2008 reference chr8

S1	S1 start	S1 stop	S2	S2 start	S2 stop
tig00000366	3	50532	Chr8	605142	655965

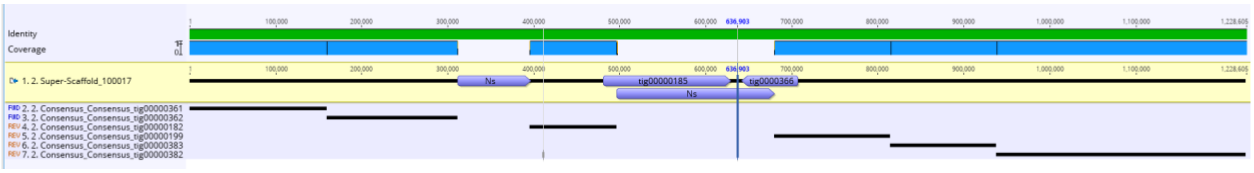

BOWLER ET AL 2008 reference CHR14 (829,358 bp)

HAPLOTYPE 1: Super-Scaffold_100033 → telomere to telomere

- 854,044 bp (length supported by PFGE)

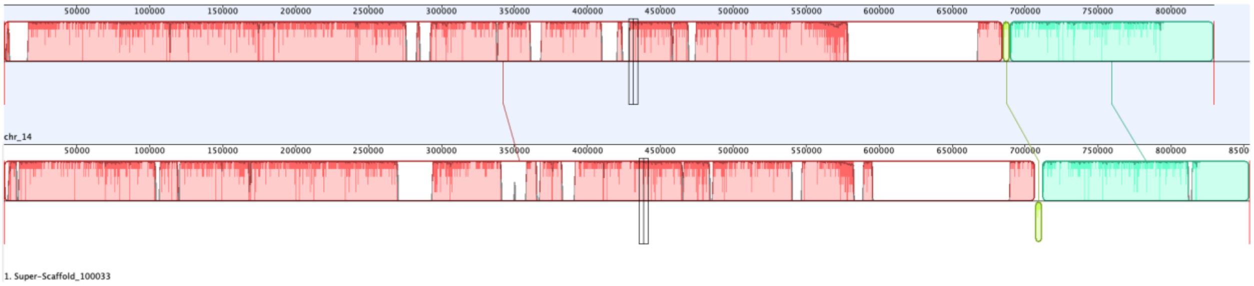

- Includes 2 canu contigs

1	594968	Consensus_Consensus_tig00000392 (-)
594969	688008	GAP 93,040 bp[1]
688009	854044	Consensus_Consensus_tig00000226 (+)

[1] GAP resolved with Consensus_Consensus_tig00000229 and Consensus_Consensus_tig00000231

Consensus_Consensus_tig00000231
- length: 70,142 bp
- this unscaffolded contig can be anchored to SS_100033 to fill in Bionano gap region
- coordinates:

S1	S1 start	S1 stop	S2	S2 start	S2 stop	% id
tig00000231	25270	2	SS_100033	689268	714561	99.5

- Consensus_Consensus_tig00000231 blasts to BOWLER ET AL 2008 reference chr14

S1	S1 start	S1 stop	S2	S2 start	S2 stop	% id
tig00000231	3	70140	Chr14	136852	197031	99.07

Consensus_Consensus_tig00000229
- length: 63,350 bp
- this unscaffolded contig can be anchored to tig00000231 to fill in Bionano gap region
- coordinates:

S1	S1 start	S1 stop	S2	S2 start	S2 stop	% id
tig00000229	44127	63350	tig00000231	70142	50926	99.5

- Consensus_Consensus_tig00000299 blasts to BOWLER ET AL 2008 reference chr14

S1	S1 start	S1 stop	S2	S2 start	S2 stop	% id
tig00000299	63348	3	Chr14	179780	246300	88.2

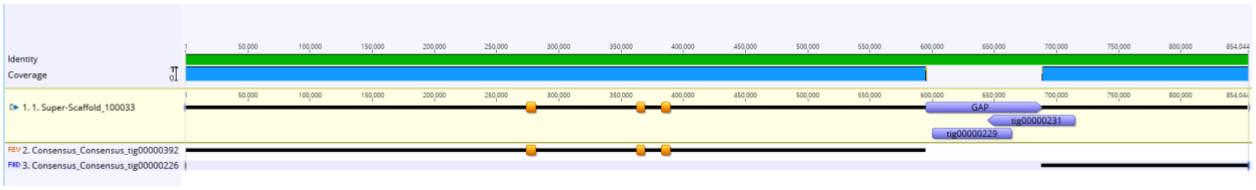

HAPLOTYPE 2: Super-Scaffold_100025 → no telomeres resolved

- 698,686 bp

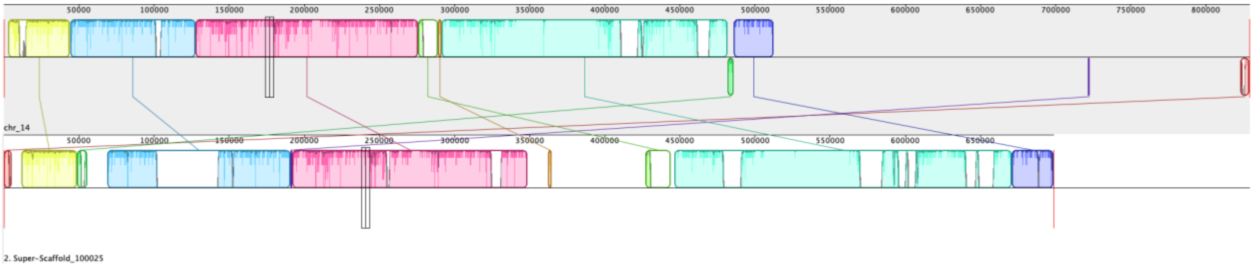

- Includes 4 canu contigs

1	122769	Consensus_Consensus_tig00000390 (+)
122770	122782	13
122783	373081	Consensus_Consensus_tig00000391 (+)
373082	373094	13
373095	569659	Consensus_Consensus_tig00000236 (-)
569660	569672	13
569673	698686	Consensus_Consensus_tig00000251 (-)

- There are two unscaffolded contigs (tig00002810 & tig00002811; see below) that align towards the end of reference chr14. SS_100025 lacks synteny to that region of reference chr14 so it is likely that those contigs should be assigned to SS_100025
- Interestingly, both contigs align to the same region of reference chr14. HAPLOTYPE 1 (SS_100033) includes sequence data homologous to that region of reference chr14.
- Neither contig can be anchored to SS_100025.

- Consensus_Consensus_ tig00002810 (84,025 bp) blasts to BOWLER ET AL 2008 reference chr14

S1	S1 start	S1 stop	S2	S2 start	S2 stop	% id
tig00002810	84021	3	Chr14	751157	829060	98.5

- Consensus_Consensus_ tig00002811 (63,756 bp) blasts to BOWLER ET AL 2008 reference chr14

S1	S1 start	S1 stop	S2	S2 start	S2 stop	% id
tig00002811	63754	3	Chr14	750432	808788	94.3
