# Supplementary material for: Re-examination of two diatom reference genomes using long-read sequencing
Source: BMC Genomics. 2021 May 24;22:379. doi: 10.1186/s12864-021-07666-3 (PMC8147415; doi:10.1186/s12864-021-07666-3)
Supplement: Supplementary file 2 — Additional file 2: Supplementary Table 1. Detailed raw read data summary for unfiltered, Albacore “pass” and filtered Oxford Nanopore long-read sequencing datasets for Thalassiosira pseudonana and Phaeodactylum tricornutum. The filtered datasets for T. pseudonana and P. tricornutum included reads ≥30 kb and ≥ 20 kb, respectively. [file 12864_2021_7666_MOESM2_ESM.pdf]

Supplementary Table 1. Detailed raw read data summary for unfiltered, Albacore "pass" and filtered Oxford Nanopore long-read sequencing datasets for *Thalassiosira pseudonana* and *Phaeodactylum tricornutum*. The filtered datasets for *T. pseudonana* and *P. tricornutum* included reads ≥30 kb and ≥20 kb, respectively.

	Thalassiosira pseudonana			*Phaeodactylum tricornutum*		
	Unfiltered Data	Albacore "pass" Data	Filtered Data	Unfiltered Data	Albacore "pass" Data	Filtered Data
General Summary						
Total bases (bp)	7,445,183,025	6,987,392,466	1,772,209,890	8,199,773,433	7,500,371,505	2,700,036,591
No. of reads	701,596	580,845	46,708	986,604	820,187	84,445
Read length N50 (bp)	20,088	20,514	37,303	18,756	19,261	32,648
Mean read length (bp)	10,611.8	12,029.7	37,942.3	8,311.1	9,144.7	31,973.9
Mean read quality	9.9	10.8	10.9	8.5	9.2	9.6
Median read length (bp)	6,755	8,578.0	36,095	3,815	4,537.0	29,187
Median read quality	10.5	11.1	11.2	8.9	9.2	9.6
Number, percentage and megabases of reads above quality cutoffs						
Q5	641,236 (91.4%)	580,845 (100.0%)	46,708 (100.0%)	917,011 (92.9%)	820,187 (100.0%)	84,445 (100.0%)
	7,416.7 Mbp	6,987.4 Mbp	1,772.2 Mbp	8,141.7 Mbp	7,500.4 Mbp	2,700.0 Mbp
Q7	580,397 (82.7%)	580,329 (99.9%)	46,676 (99.9%)	819,256 (83.0%)	819,380 (99.9%)	84,445 (100.0%)
	6,982.1 Mbp	6,982.1 Mbp	1,771.0 Mbp	7,513.9 Mbp	7,494.6 Mbp	2,700.0 Mbp
Q10	391,138 (55.7%)	391,138 (67.3%)	32,875 (70.4%)	189,235 (19.2%)	195,528 (23.8%)	25,313 (30.0%)
	4,788.0 Mbp	4,788.0 Mbp	1,251.2 Mbp	1,681.4 Mbp	1,694.8 Mbp	790.5 Mbp
Q12	192,403 (27.4%)	192,403 (33.1%)	16,143 (34.6%)	6,828 (0.7%)	7,207 (0.9%)	786 (0.9%)
	2,321.3 Mbp	2,321.3 Mbp	614.2 Mbp	55.5 Mbp	56.4 Mbp	23.6 Mbp
Q15	111 (0.0%)	111 (0.0%)	0 (0.0%)	0 (0.0%)	1 (0.0%)	0 (0.0%)
	0.3 Mbp	0.3 Mbp	0.0 Mbp	0.0 Mbp	0.0 Mbp	0.0 Mbp
Top 5 highest mean base-call quality scores and read lengths						
1	16.3 (712)	16.3 (712)	15.0 (36,789)	14.9 (1,287)	15.1 (769)	14.2 (25,833)
2	16.1 (776)	16.1 (776)	14.9 (38,761)	14.9 (7,981)	15.0 (725)	14.1 (38,993)
3	16.1 (696)	16.1 (696)	14.8 (34,635)	14.8 (2,594)	15.0 (1,256)	14.0 (34,753)
4	15.9 (495)	15.9 (495)	14.7 (32,641)	14.7 (757)	14.9 (7,948)	13.9 (33,295)
5	15.9 (2,188)	15.9 (2,188)	14.6 (45,800)	14.6 (2,052)	14.9 (2,576)	13.9 (22,405)
Top 5 longest reads and mean basecall quality score						
1	122,142 Mbp (12.3)	122,142 Mbp (12.3)	122,142 Mbp (12.3)	282,534 Mbp (6.4)	140,471 Mbp (8.4)	140,471 Mbp (8.4)
2	110,728 Mbp (8.5)	110,728 Mbp (8.5)	110,728 Mbp (8.5)	140,502 Mbp (8.4)	139,006 Mbp (9.1)	139,006 Mbp (9.1)
3	99,505 Mbp (10.4)	99,505 Mbp (10.4)	995,05 Mbp (10.4)	139,039 Mbp (9.1)	125,792 Mbp (8.5)	125,792 Mbp (8.5)
4	97,967 Mbp (9.1)	97,967 Mbp (9.1)	97,967 Mbp (9.1)	125,837 Mbp (8.5)	125,267 Mbp (9.9)	125,267 Mbp (9.9)
5	97,308 Mbp (10.2)	97,308 Mbp (10.2)	97,308 Mbp (10.2)	125,299 Mbp (9.9)	120,285 Mbp (9.5)	120,285 Mbp (9.5)
