# Supplementary material for: Re-examination of two diatom reference genomes using long-read sequencing
Source: BMC Genomics. 2021 May 24;22:379. doi: 10.1186/s12864-021-07666-3 (PMC8147415; doi:10.1186/s12864-021-07666-3)
Supplement: Supplementary file 3 — Additional file 3: Supplementary Table 2. Assembly statistics for various polishing iterations of the de novo long-read derived genomes for Thalassiosira pseudonana and Phaeodactylum tricornutum. Asterisks indicate the final working assembly for each species. [file 12864_2021_7666_MOESM3_ESM.pdf]

Supplementary Table 2. Assembly statistics for various polishing iterations of the de novo long-read derived genomes for *Thalassiosira pseudonana* and *Phaeodactylum tricornutum*. Asterisks indicate the final working assembly for each species.

	Assembly	Total length (Mbp)	Read depth coverage	No. contigs	Largest contig (Mbp)	Contig N50 (Mbp)	Contig L50	G+C content (%)	% identity to reference	Busco completeness	ALE score
Phaeodactylum tricornutum	Canu unpolished	56.5	40x	291	2.49	0.25	43	48.7	98.5	C:29.1%	-1,142,001,685
	Canu + Racon 2x	56.9	40x	291	2.50	0.25	43	48.7	98.8	C:54.8%	-1,030,248,077
	Canu + Racon 2x + Nanopolish	57.0	40x	291	2.51	0.25	43	48.7	99.1	C:77.3%	-808,873,077
	Canu + Racon 2x + Nanopolish + Pilon 28x	57.0	40x	291	2.51	0.25	43	48.7	99.3	C:85.4%	-734,959,595
	Flye unpolished	33.8	72x	204	1.68	0.37	24	48.4	98.4	C:22.1%	-1,157,066,360
	Flye + Racon 2x	33.5	72x	196	1.66	0.36	24	48.6	98.7	C:49.2%	-1,002,003,578
	Flye + Racon 2x + Nanopolish	33.4	72x	196	1.66	0.36	24	48.7	99.0	C:70.6%	-848,932,570
	Flye + Racon 2x + Nanopolish + Pilon 19x*	33.5	72x	196	1.66	0.36	24	48.7	99.1	C:80.9%	-781,367,384
Thalassiosira pseudonana	Canu unpolished	46.8	40x	222	2.75	0.97	14	46.8	98.4	C:25.7%	-1,789,994,248
	Canu + Racon 2x	47.0	40x	222	2.78	0.99	14	46.8	98.9	C:46.2%	-1,625,885,182
	Canu + Racon 2x + Nanopolish	47.4	40x	222	2.77	0.98	14	46.9	99.3	C:76.9%	-976,974,396
	Canu + Racon 2x + Nanopolish + Pilon 18x	47.3	40x	222	2.77	0.98	14	46.9	99.4	C:79.2%	-1,238,092,187
	Flye unpolished	34.0	48x	57	2.77	1.38	8	46.8	98.8	C:31.7%	-1,341,668,132
	Flye + Racon 2x	33.8	48x	50	2.76	1.38	8	46.8	98.9	C:49.2%	-1,302,425,801
	Flye + Racon 2x + Nanopolish	33.8	48x	50	2.76	1.38	8	46.9	99.3	C:77.2%	-1,106,746,104
	Flye + Racon 2x + Nanopolish + Pilon 20x*	33.8	48x	52	2.76	1.38	8	47.0	99.4	C:80.6%	-1,047,071,217
