# Supplementary material for: Re-examination of two diatom reference genomes using long-read sequencing
Source: BMC Genomics. 2021 May 24;22:379. doi: 10.1186/s12864-021-07666-3 (PMC8147415; doi:10.1186/s12864-021-07666-3)
Supplement: Supplementary file 6 — Additional file 6: Supplementary Table 5. Structural variation between the polished de novo Canu and Flye assemblies and the reference genomes for Phaeodactylum tricornutum and Thalassiosira pseudonana. [file 12864_2021_7666_MOESM6_ESM.pdf]

Supplementary Table 5. Structural variation between the polished de novo Canu and Flye assemblies and the reference genomes for *Phaeodactylum tricornutum* and *Thalassiosira pseudonana.*

		Phaeodactylum tricornutum		*Thalassiosira pseudonana*	
		Canu		Flye	
Variant category	Size range	Count	Total bp	Count	Total bp
Insertion	25-50 bp	141	4,573	55	1,730
	50-500 bp	130	28,450	79	16,650
	500-4,000 bp	147	251,899	64	99,587
	4,000-10,000 bp	187	1,188,029	17	103,180
	10,000-50,000 bp	19	286,290	0	0
	Total	624	1,759,241	215	221,147
	25-50 bp	136	4,350	292	9,675
Deletion	50-500 bp	136	25,699	78	10,359
	500-4,000 bp	65	92,862	18	33,345
	4,000-10,000 bp	33	182,423	7	41,834
	10,000-50,000 bp	3	33,555	0	0
	Total	373	338,889	395	95,213
	25-50 bp	0	0	0	0
Tandem expansion	50-500 bp	28	7,732	20	5,378
	500-4,000 bp	100	156,993	83	138,699
	4,000-10,000 bp	38	349,511	23	141,259
	10,000-50,000 bp	22	356,201	18	260,192
	Total	188	870,437	144	545,528
	25-50 bp	0	0	0	0
Tandem contraction	50-500 bp	7	1,081	10	1,975
	500-4,000 bp	4	5,601	8	14,718
	4,000-10,000 bp	12	89,581	1	4,055
	10,000-50,000 bp	7	113,143	1	19,288
	Total	30	209,406	20	40,036
	25-50 bp	5	177	6	210
Repeat expansion	50-500 bp	46	9,861	36	7,752
	500-4,000 bp	14	120,371	29	50,779
	4,000-10,000 bp	103	332,319	7	46,568
	10,000-50,000 bp	28	404,521	0	0
	Total	196	867,249	78	105,380
	25-50 bp	6	194	6	222
Repeat contraction	50-500 bp	44	9,283	39	9,338
	500-4,000 bp	16	90,900	25	44,439
	4,000-10,000 bp	70	166,181	9	53,473
	10,000-50,000 bp	22	372,826	4	87,343
	Total	158	639,384	83	194,815
		1,569	4.68 Mbp	935	1.20 Mbp
Total for all variants		1,281	4.68 Mbp	576	1.19 Mbp
