# Supplementary material for: Re-examination of two diatom reference genomes using long-read sequencing
Source: BMC Genomics. 2021 May 24;22:379. doi: 10.1186/s12864-021-07666-3 (PMC8147415; doi:10.1186/s12864-021-07666-3)
Supplement: Supplementary file 9 — Additional file 9: Supplementary Table 8. Comparison of RepeatMasker determined repetitive content for the final polished long-read working assembly and the reference genome for Phaeodactylum tricornutum. Note that the numbers reported for LTR elements include full-length, nested and decaying elements. [file 12864_2021_7666_MOESM9_ESM.pdf]

Supplementary Table 8. Comparison of RepeatMasker determined repetitive content for the final polished long-read working assembly and the reference genome for *Phaeodactylum tricornutum*. Note that the numbers reported for LTR elements include full-length, nested and decaying elements.

	Canu			Reference		
	total length: 57.0 Mbp<br>contigs:291<br>bases masked: 11.3 Mbp (19.9%)			total length: 27.4 Mbp<br>contigs: 88<br>bases masked: 2.22 Mbp (8.1%)		
	number of elements	length occupied (bp)	% of sequence	number of elements	length occupied (bp)	% of sequence
SINEs:	42	136,946	0.2	0	0	0
ALUs	0	0	0	0	0	0
MIRs	0	0	0	0	0	0
LINEs:	90	145,343	0.3	0	0	0
LINE1	0	0	0	0	0	0
LINE2	0	0	0	0	0	0
L3/CR1	0	0	0	0	0	0
LTR elements:	4,703	8,186,677	14.4	1,383	1,574,225	5.7
ERVL	0	0	0	0	0	0
ERVL-MaLRs	0	0	0	0	0	0
ERV_classI	0	0	0	0	0	0
ERV_classII	7	7,321	1	0	0	0
DNA elements:	770	581,421	1.0	323	192,701	0.7
hAT-Charlie	225	89,154	0.2	73	19,894	0.1
TcMar-Tigger	0	0	0	0	0	0
Unclassified:	4,325	2,110,412	3.7	1,745	447,030	1.6
Total interspersed repeats:		11,160,799	19.6		2,213,956	8.1
Small RNA:	20	66,328	0.1	0	0	0
Satellites:	37	4,179	0.01	0	0	0
Simple repeats:	90	111,515	0.2	35	5,794	0.02
Low complexity:	0	0	0	0	0	0
