# Supplementary material for: Re-examination of two diatom reference genomes using long-read sequencing
Source: BMC Genomics. 2021 May 24;22:379. doi: 10.1186/s12864-021-07666-3 (PMC8147415; doi:10.1186/s12864-021-07666-3)
Supplement: Supplementary file 10 — Additional file 10: Supplementary Table 9. Comparison of RepeatMasker determined repetitive content for the final polished long-read working assembly and the reference genome for Thalassiosira pseudonana. Note that the numbers reported for LTR elements include full-length, nested and decaying elements. [file 12864_2021_7666_MOESM10_ESM.pdf]

Supplementary Table 9. Comparison of RepeatMasker determined repetitive content for the final polished long-read working assembly and the reference genome for *Thalassiosira pseudonana*. Note that the numbers reported for LTR elements include full-length, nested and decaying elements.

	Flye			Reference		
	total length: 33.8 Mbp<br>contigs: 52<br>bases masked: 2.55 Mbp (7.6%)			length: 32.4 Mbp<br>contigs: 64<br>bases masked: 1.03 Mbp (3.2%)		
	number of elements	length occupied (bp)	% of sequence	number of elements	length occupied (bp)	% of sequence
SINEs:	0	0	0	0	0	0
ALUs	0	0	0	0	0	0
MIRs	0	0	0	0	0	0
LINEs:	222	223,759	0.7	175	86,304	0.3
LINE1	0	0	0	0	0	0
LINE2	0	0	0	0	0	0
L3/CR1	0	0	0	0	0	0
LTR elements:	808	737,184	2.2	545	245,854	0.8
ERVL	0	0	0	0	0	0
ERVL-MaLRs	0	0	0	0	0	0
ERV_classI	0	0	0	0	0	0
ERV_classII	0	0	0	0	0	0
DNA elements:	454	314,007	0.9	329	113,973	0.4
hAT-Charlie	0	0	0	0	0	0
TcMar-Tigger	0	0	0	0	0	0
Unclassified:	2,630	1,118,054	3.3	1542	521,581	1.61
Total interspersed		2,393,004	7.1		967,712	3.0
Small RNA:	43	72,753	0.2	0	0	0
Satellites:	95	70,767	0.2	40	24,498	0.1
Simple repeats:	80	17,638	0.1	92	43,971	0.1
Low complexity:	0	0	0	0	0	0
