# Supplementary material for: Re-examination of two diatom reference genomes using long-read sequencing
Source: BMC Genomics. 2021 May 24;22:379. doi: 10.1186/s12864-021-07666-3 (PMC8147415; doi:10.1186/s12864-021-07666-3)
Supplement: Supplementary file 14 — Additional file 14: Supplementary Figure 1. Workflow of sample preparation, MinION sequencing, de novo genome assembly and downstream analyses, including methods to compare the de novo long-read reference assemblies and gene prediction. Supplementary Figure 2. Bivariate scatterplots showing the relationship of MinION read lengths and average Phred read quality scores for Phaeodactylum tricornutum (A & B) and Thalassiosira pseudonana (C & D). The unfiltered datasets (A & C) include all generated MinION data and the filtered datasets (B & D) include a subset of reads filtered by length and quality. Supplementary Figure 3. Pulsed-field gel electrophoresis of P. tricornutum (CCMP632) DNA using settings to optimize resolution of large (A) and small (B) fragments. The red arrowheads (<) indicate potential chromosome-sized fragments. Ladders for sizing fragments include Saccharomyces cerevisiae (Sc) and Hansenula wingei (Hw). Lanes labelled 1–4 and 8–13 are not relevant to this study. Supplementary Figure 4. Relationship between 16,491 predicted protein models from the Flye Thalassiosira pseudonana assembly to reference protein set and the matches to known proteins of the new predicted protein models (2010). Supplementary Figure 5. PloidyNGS plot of the frequency of the two most abundant alleles in the Phaeodactylum tricornutum genome indicates that it is a diploid organism. Supplementary Figure 6. Stacked histograms showing the different categories of P. tricornutum haplotypes supported by Bionano hybrid scaffolding. Characterizations are based on a total of 66 haplotypes expected based on a diploid genome with 33 chromosomes as estimated for the reference genome. Supplementary Figure 7. ‘Hybrid-hybrid’ Bionano-Canu super-scaffolds that may represent mis-assemblies owing to segmental duplications (A & B) or LTR-RT insertions (C) located at the ends of Canu contigs. Mauve [81] schematics of the syntenic regions between a Bionano-Canu super-scaffold and the reference chromo [file 12864_2021_7666_MOESM14_ESM.pdf]

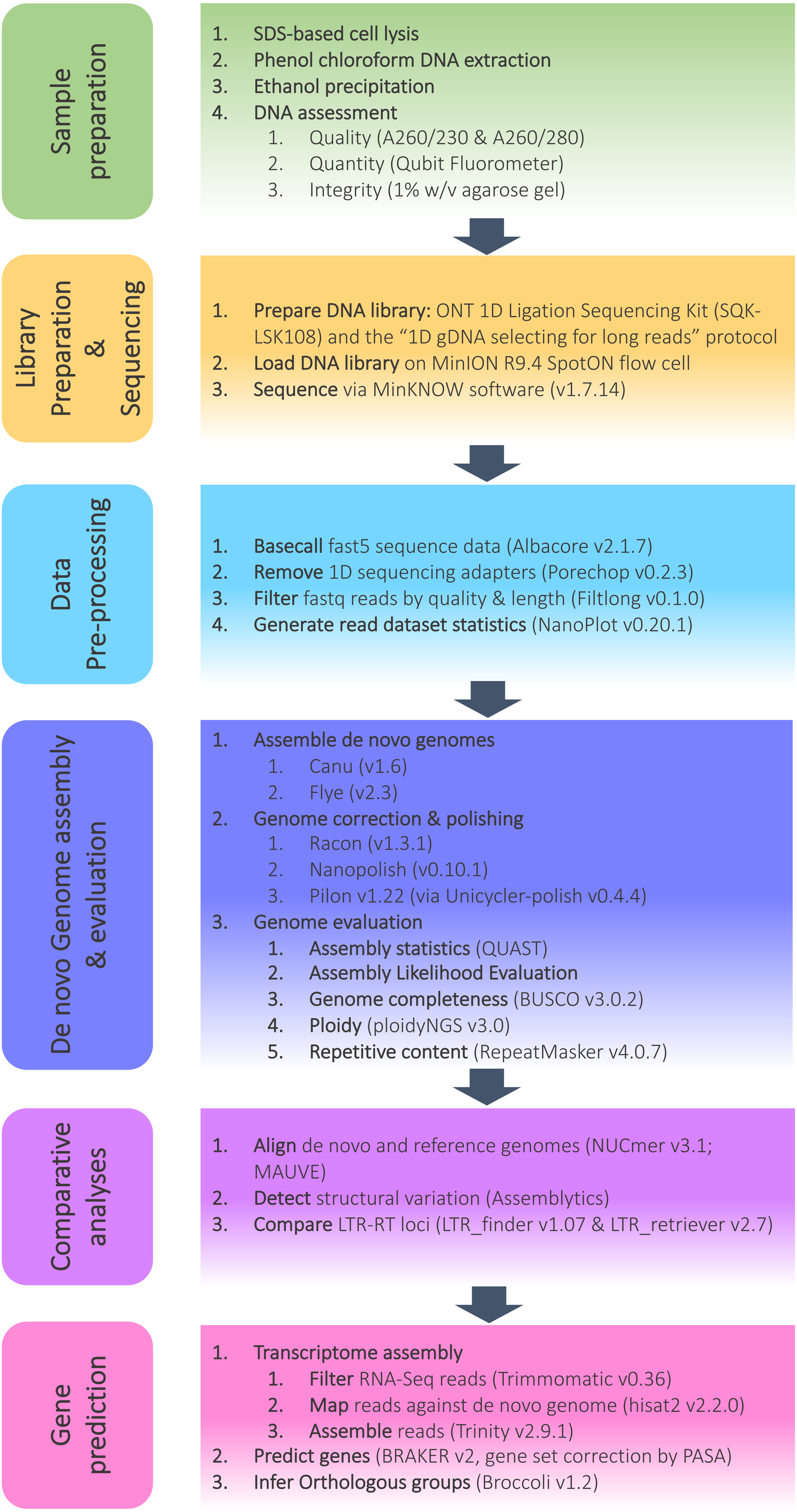

Supplementary Figure 1

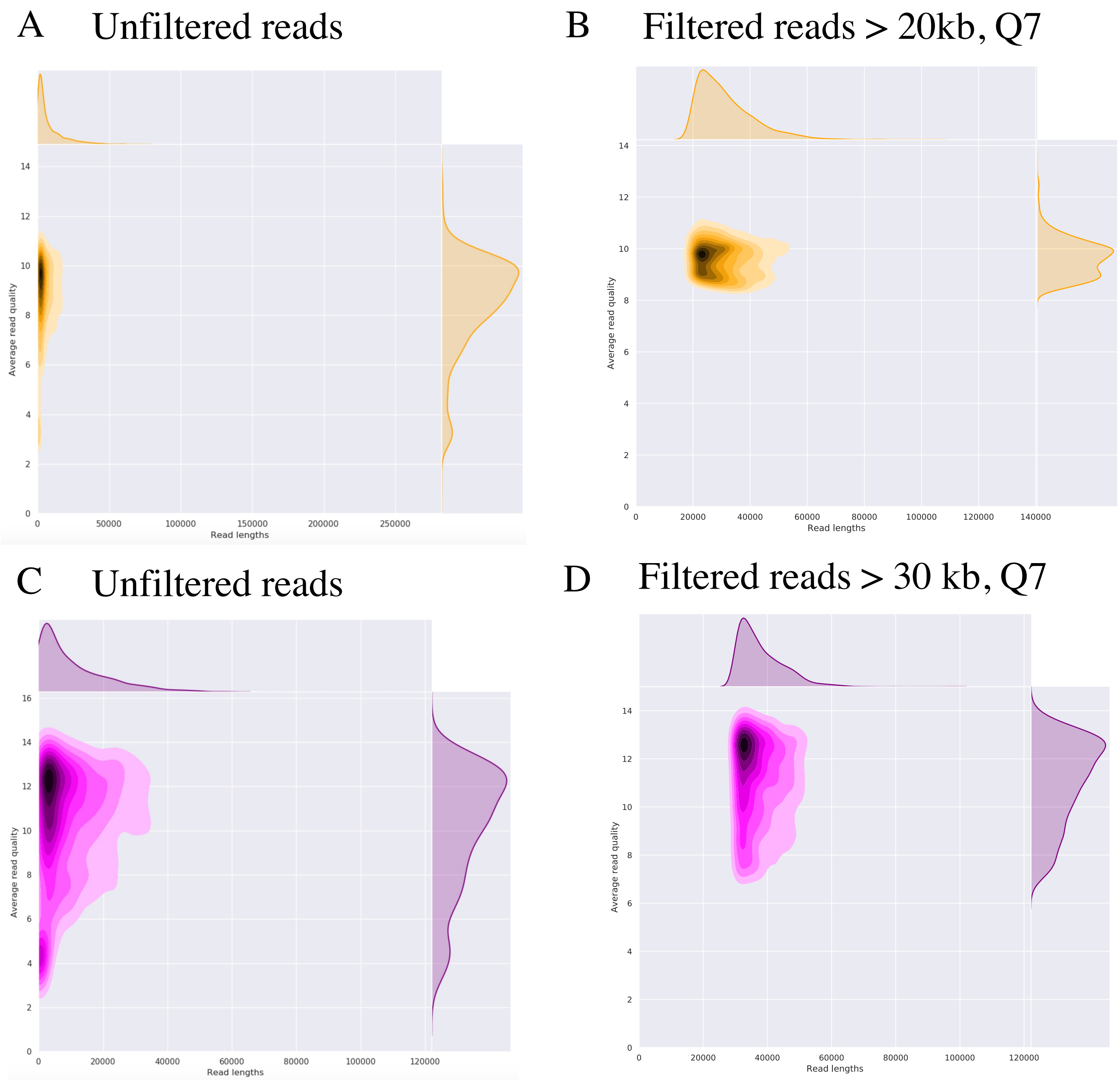

Supplementary Figure 2

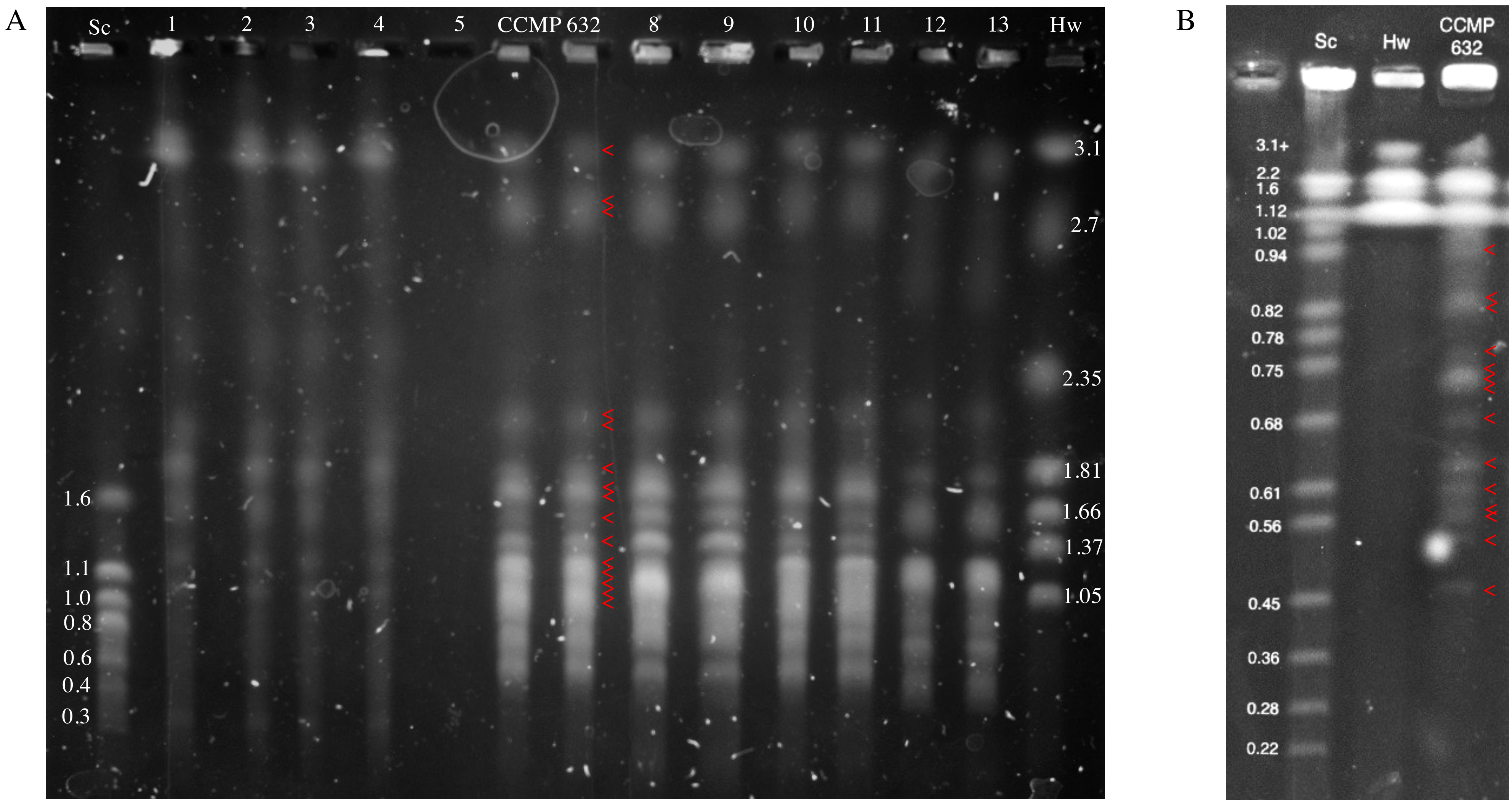

Supplementary Figure 3

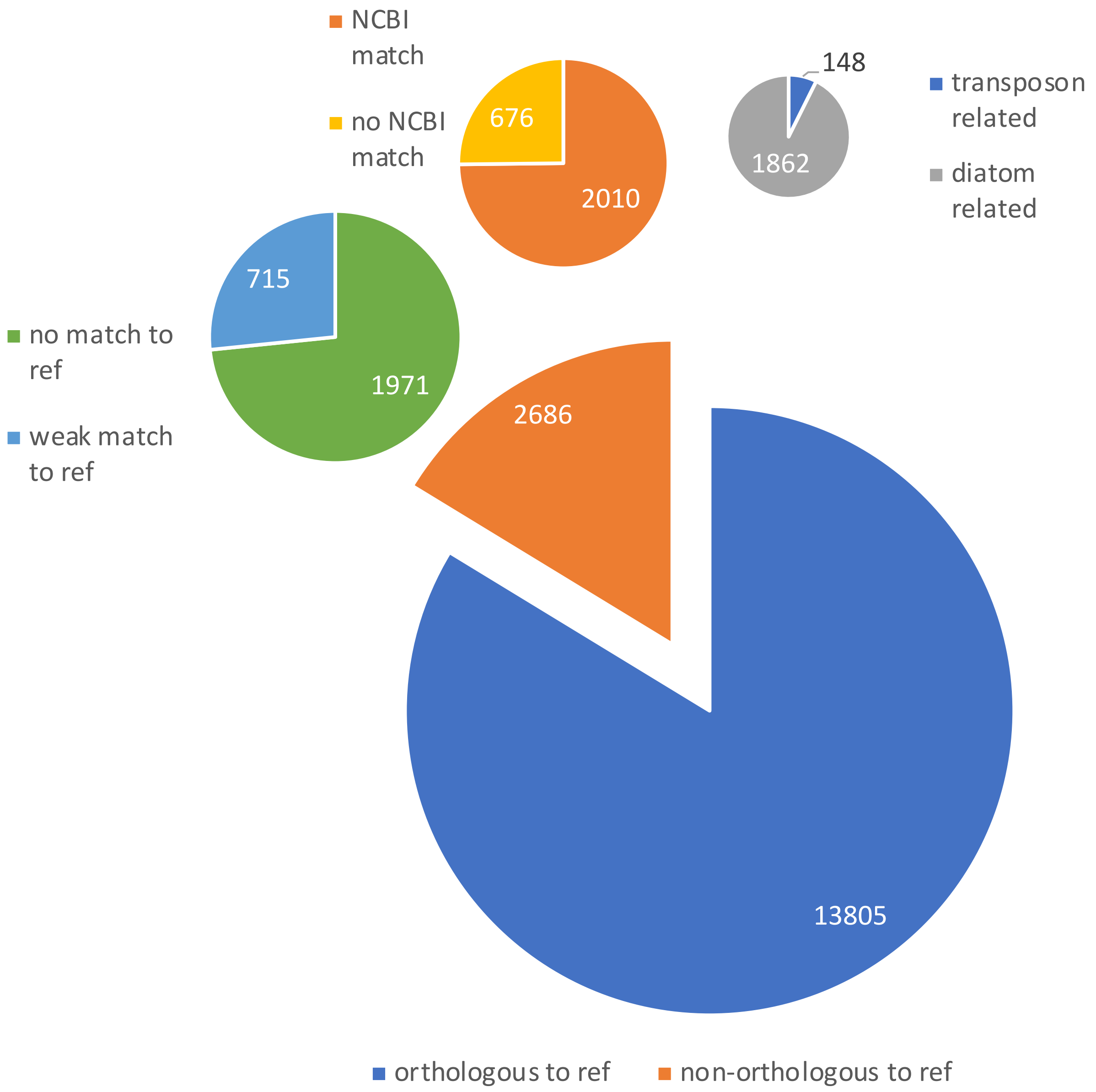

Supplementary Figure 4

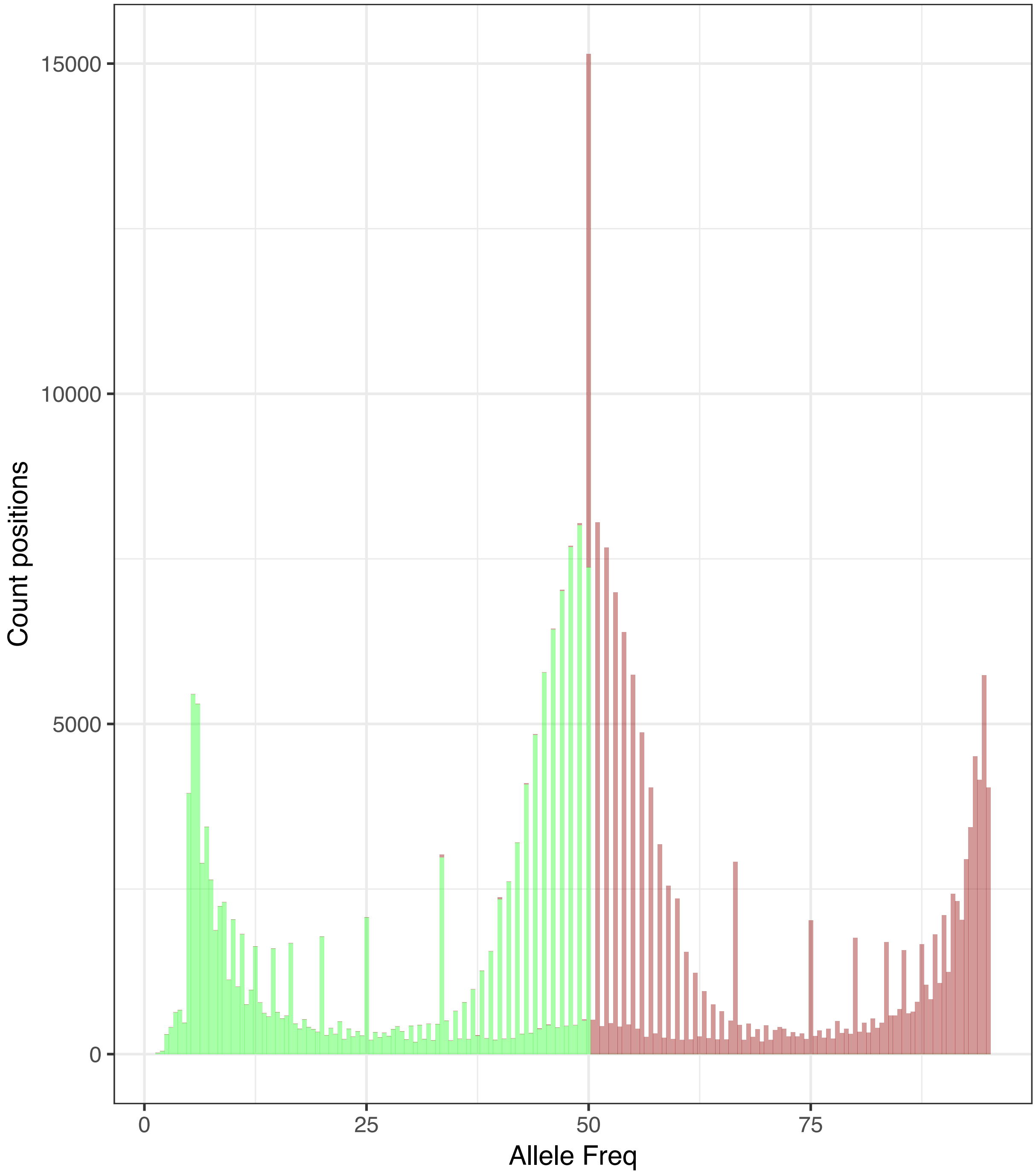

Supplementary Figure 5

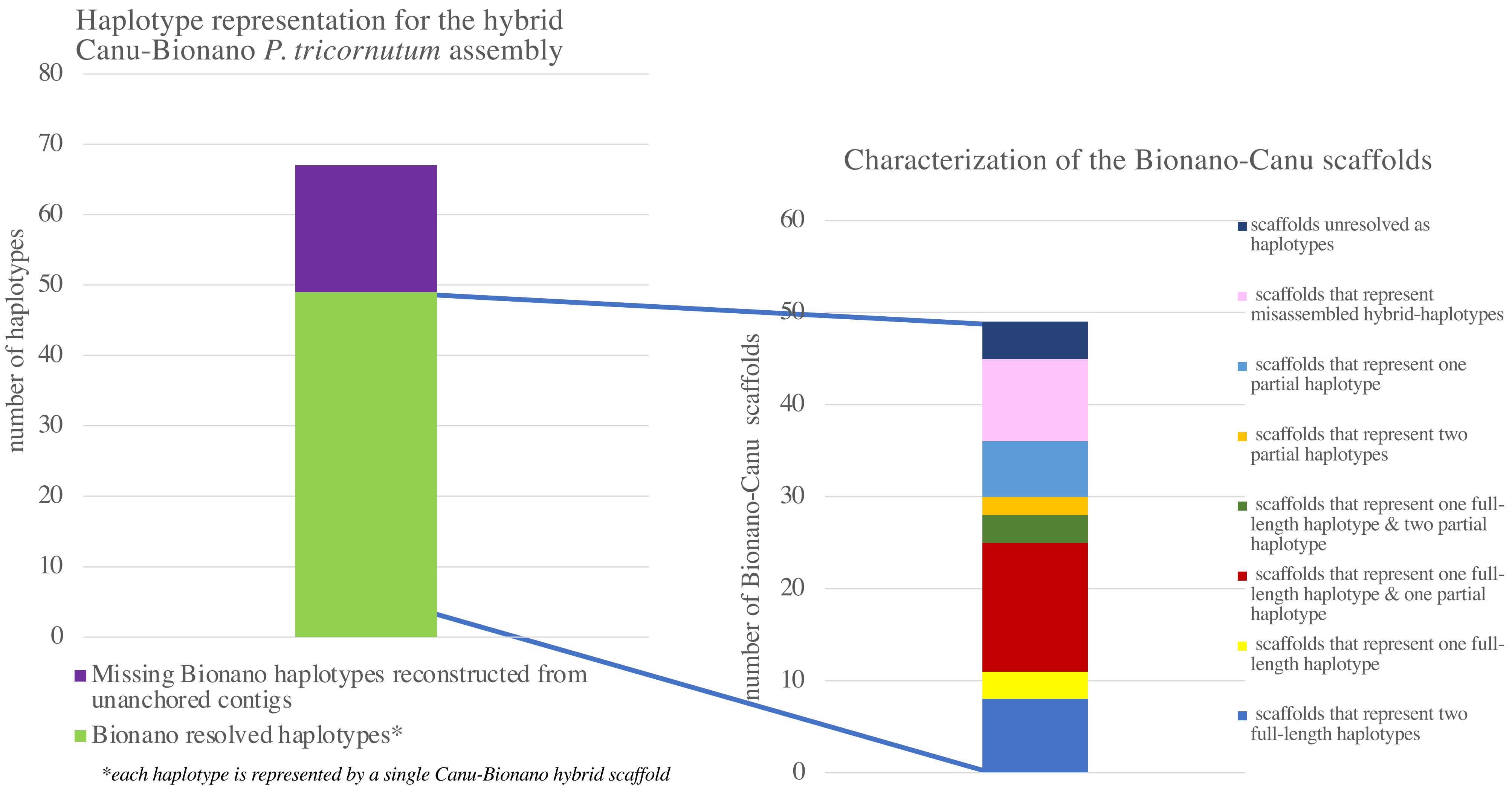

Supplementary Figure 6

Super-Scaffold_100002

chr28-chr7-chr13 hybrid contigs are erroneously linked owing to segmental duplications between those chromosome pairs

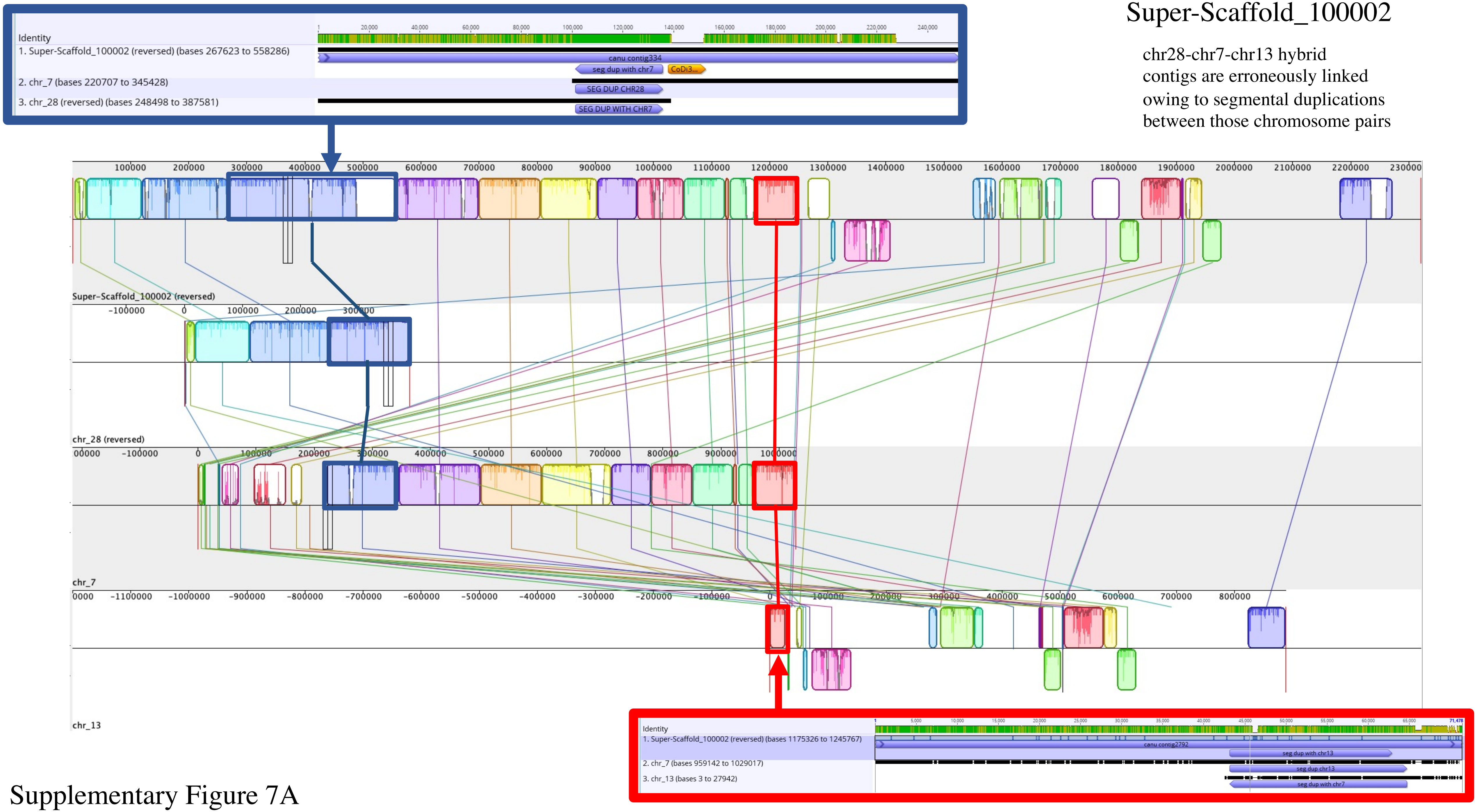

Supplementary Figure 7A

Super-Scaffold_100022

chr24-chr29 hybrid
contigs were erroneously linked owing to segmental duplications
between those chromosome pairs

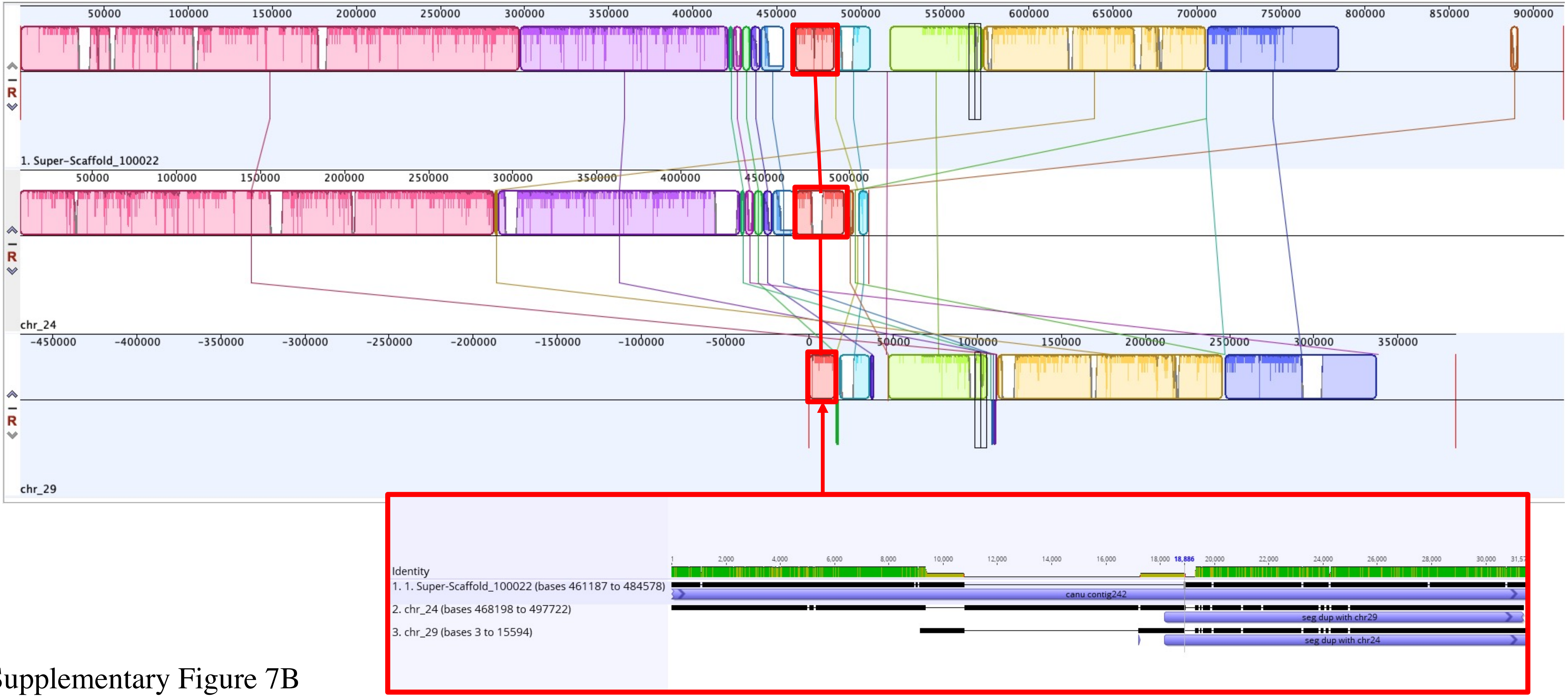

Supplementary Figure 7B

Super-Scaffold_100015

chr23-chr19 hybrid
contigs were erroneously linked owing to CoDi2.1 repetitive elements

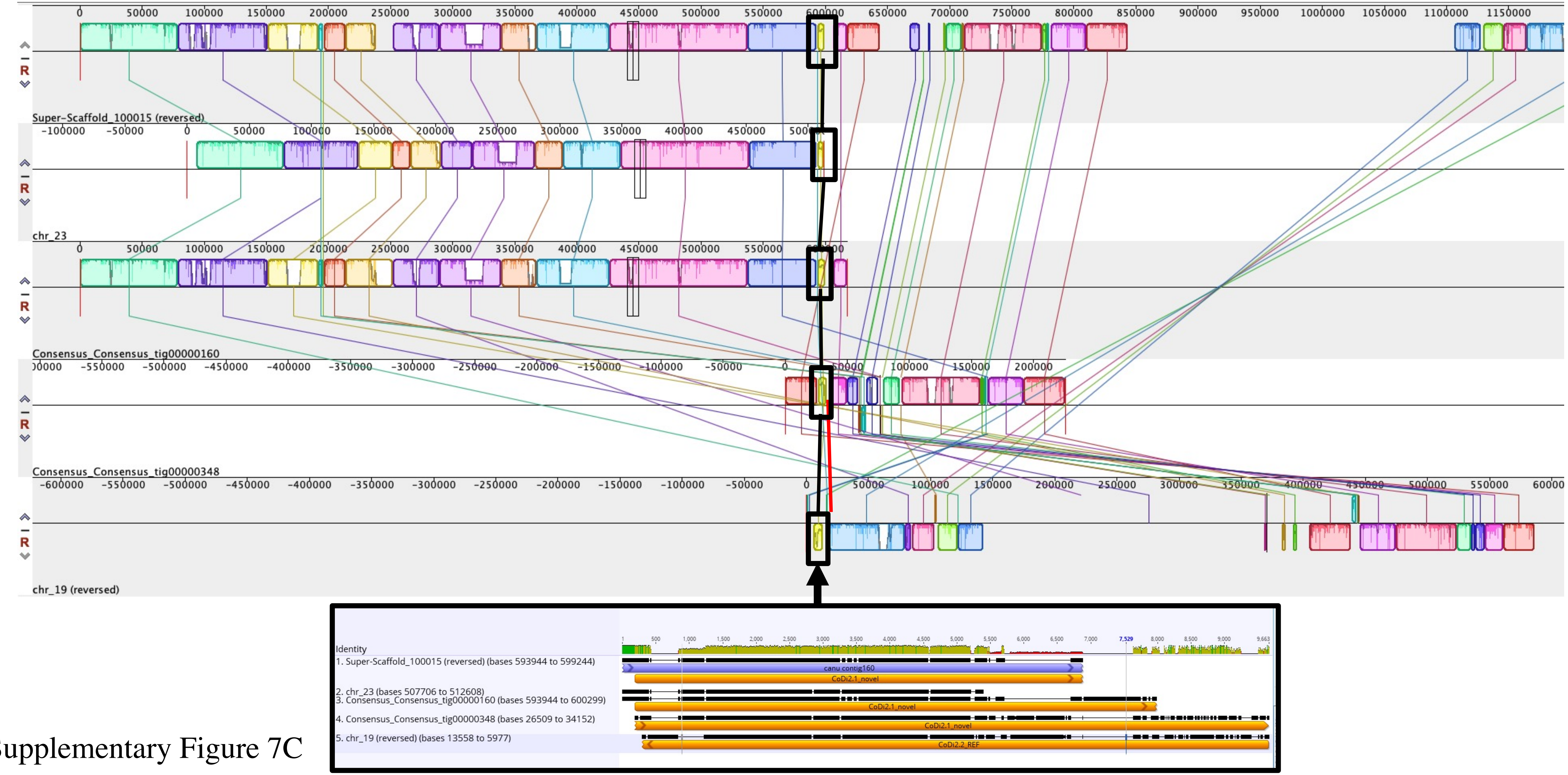

Supplementary Figure 7C

Super-Scaffold_100016

chr17—chr27 hybrid

Reference chr17 and chr27 blasted against Super-Scaffold_100016

	Description	Max Score	Total Score	Query Cover	E value	Per. Ident	Acc. Len	Accession
☑	chr17	1.904e+05	1.224e+06	54%	0.0	98.90%	703943	Query_59208
☑	chr27	95960	2.698e+05	12%	0.0	99.28%	404298	Query_59209

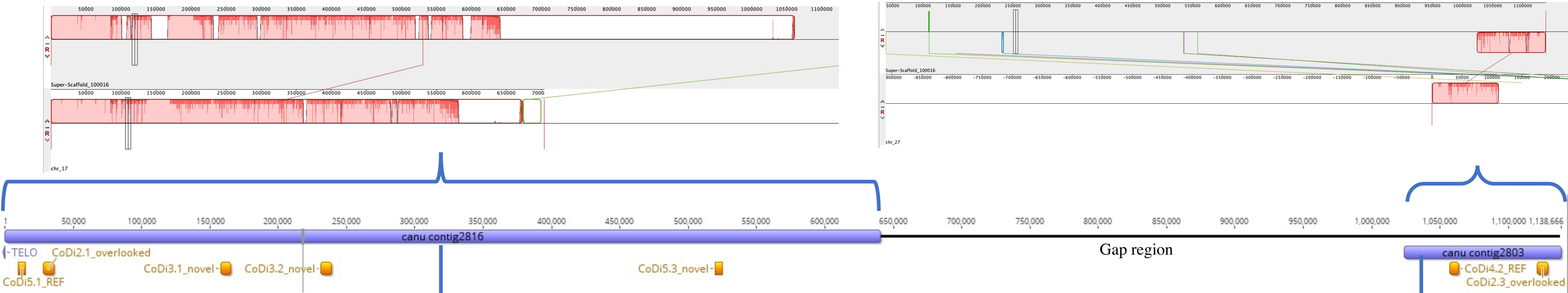

Portions of Canu contig2816 blasts with high query coverage to chr17. The small sequence that aligns to chr27 is part of the CoDi3.2 locus.

	Description	Max Score	Total Score	Query Cover	E value	Per. Ident	Acc. Len	Accession
☑	chr17	1.904e+05	1.207e+06	95%	0.0	98.90%	703943	Query_65036
☑	chr27	4948	57095	5%	0.0	98.44%	404298	Query_65037

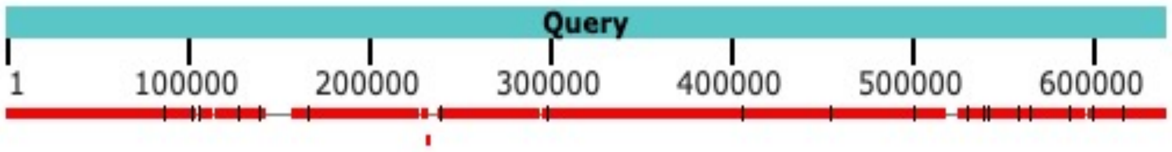

Almost the entirety of Canu contig2803 blasts strongly to chr27

	Description	Max Score	Total Score	Query Cover	E value	Per. Ident	Acc. Len	Accession
☑	chr27	95960	2.127e+05	97%	0.0	99.28%	404298	Query_47591
☑	chr17	2302	16455	11%	0.0	95.86%	703943	Query_47590

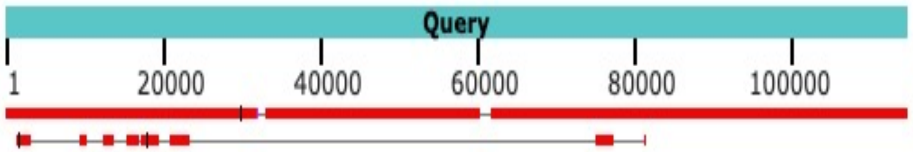

Supplementary Figure 8A

Super-Scaffold_100007

chr7—chr22 hybrid

Reference chr22 and chr7 blasted against Super-Scaffold_100007

Description	Max Score	Total Score	Query Cover	E value	Per. Ident	Acc. Len	Accession
chr22	1.510e+05	1.114e+06	29%	0.0	99.41%	591336	Query_3346
chr7	1.102e+05	6.799e+05	18%	0.0	99.58%	1029019	Query_3347

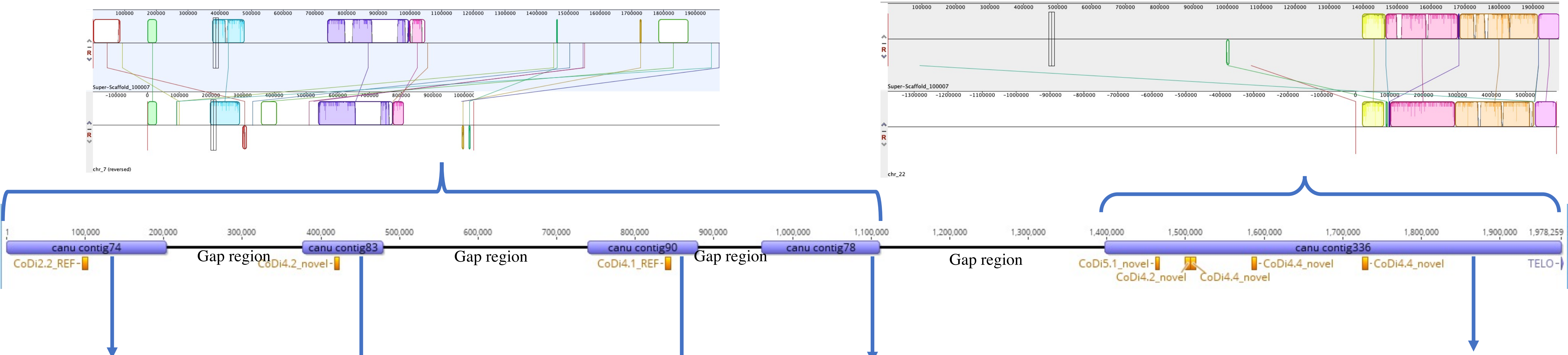

Description	Total Score	Query Cover	Per. Ident	Acc. Len
chr7	98817	25%	99.99%	1029019
chr22	21159	6%	95.28%	591336

Description	Total Score	Query Cover	Per. Ident	Acc. Len
chr7	1.731e+05	95%	99.42%	1029019
chr22	1169	2%	76.06%	591336

Description	Total Score	Query Cover	Per. Ident	Acc. Len
chr7	2.370e+05	91%	99.58%	1029019
chr22	26125	10%	95.30%	591336

Description	Total Score	Query Cover	Per. Ident	Acc. Len
chr7	1.289e+05	44%	98.87%	1029019
chr22	1703	0%	89.62%	591336

Almost the entirety of Canu contig336 is homologous to chr22.

Description	Max Score	Total Score	Query Cover	E value	Per. Ident	Acc. Len	Accession
chr22	1.510e+05	1.064e+06	94%	0.0	99.41%	591336	Query_30794
chr7	7101	42091	5%	0.0	93.55%	1029019	Query_30795

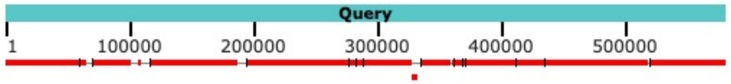

Supplementary Figure 8B

Super-Scaffold_100014
chr27—chr17 hybrid

Reference chr27 and chr17 blasted against Super-Scaffold_100014

	Description	Total Score	Query Cover	E value	Per. Ident	Acc. Len
☑	chr27	7.802e+05	37%	0.0	99.03%	404298
☑	chr17	6.373e+05	28%	0.0	99.42%	703943

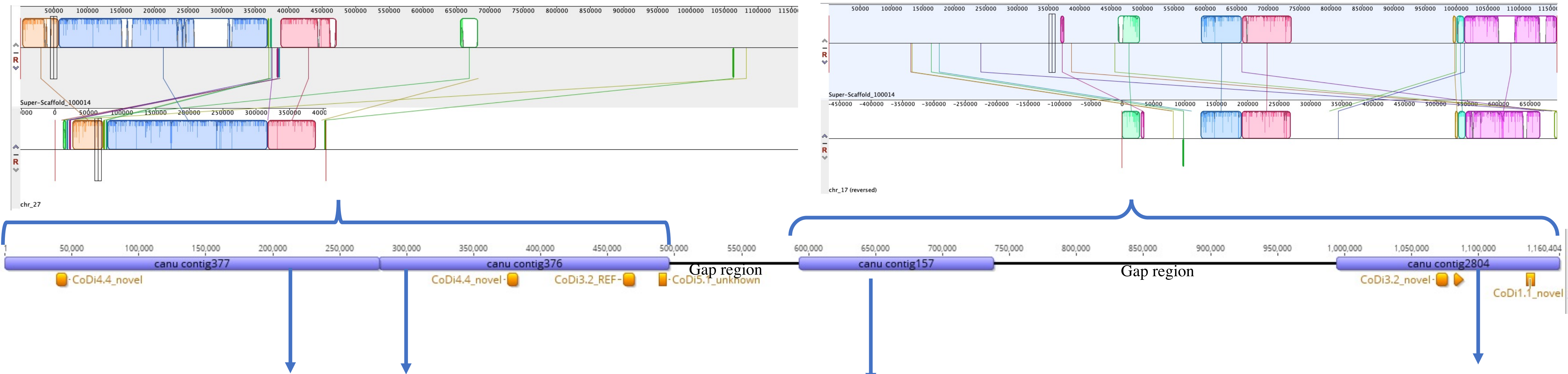

Canu contig377 and contig 376 have 99% identity to chr27 with a qcov against chr2 of 88% and 80%, respectively. Contig377 has a qcov to chr17 of 3% while the qcov to chr17 for contig 376 is 23%.

Contig377

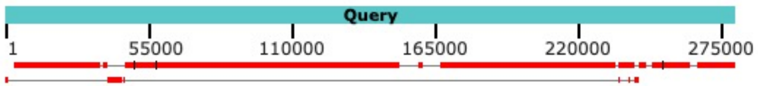

Contig376

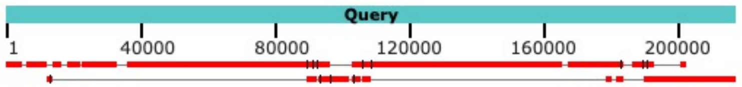

Canu contig157 blasts to chr17 along its entire length.

Description	Total Score	Query Cover	Per. Ident	Acc. Len
chr17	2.716e+05	98%	99.42%	703943
chr27	3552	2%	82.54%	404298

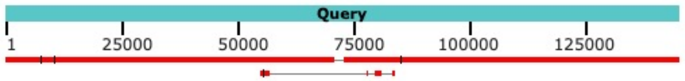

Canu contig2804 blasts to chr17 along the majority of its length.

Description	Total Score	Query Cover	Per. Ident	Acc. Len
chr17	2.376e+05	80%	97.50%	703943
chr27	20693	5%	99.14%	404298

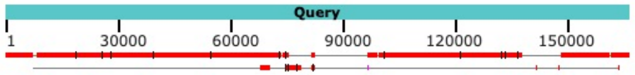

Supplementary Figure 8C

Super-Scaffold_100006
chr15—chr4 hybrid

Reference chr4 and chr15 blasted against Super-Scaffold_100006

	Description	Max Score	Total Score	Query Cover	E value	Per. Ident	Acc. Len
☑	chr4	4.301e+05	2.358e+06	53%	0.0	99.34%	1360148
☑	chr15	2.161e+05	1.316e+06	30%	0.0	99.15%	814910

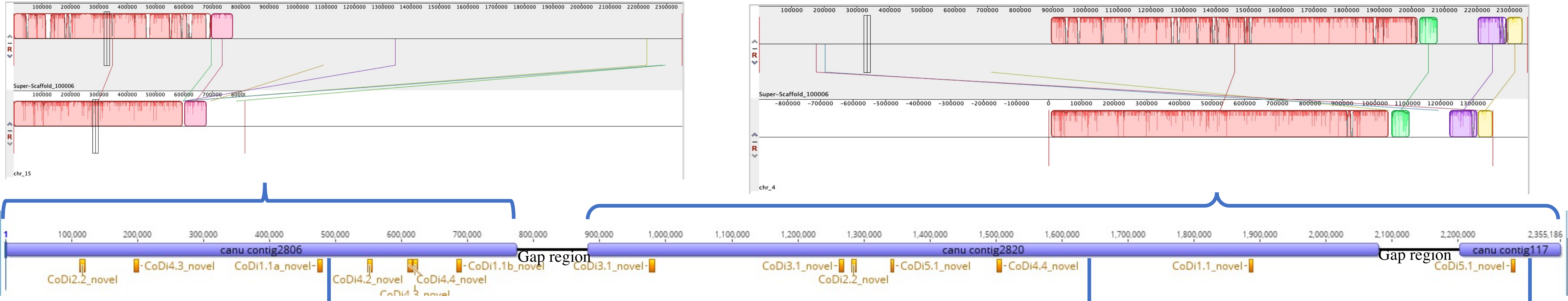

Almost the entirety of Canu contig2806 blasts to chr15 with low query coverage to chr4.

	Description	Max Score	Total Score	Query Cover	Per. Ident	Acc. Len
☑	chr15	2.161e+05	1.251e+06	87%	99.15%	814910
☑	chr4	2892	39615	3%	80.67%	1360148

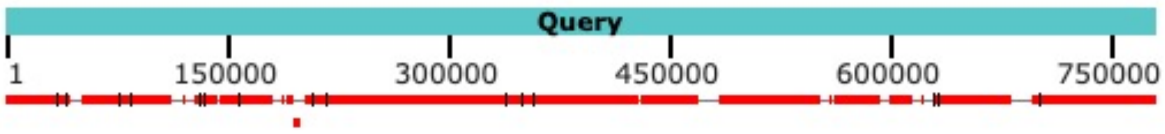

Almost the entirety of Canu contig2820 blasts to chr4 with low query coverage to chr15.

	Description	Total Score	Query Cover	Per. Ident	Acc. Len
☑	chr4	2.054e+06	92%	99.34%	1360148
☑	chr15	44505	1%	82.47%	814910

Almost the entirety of Canu contig117 blasts to chr4 with low query coverage to chr15.

	Description	Total Score	Query Cover	Per. Ident	Acc. Len
☑	chr4	2.645e+05	87%	98.96%	1360148
☑	chr15	20733	7%	82.79%	814910

Supplementary Figure 8D

Super-Scaffold_100008

chr13—chr18 hybrid

Reference chr13 and chr18 blasted against Super-Scaffold_100008

	Description	Common Name	Max Score	Total Score	Query Cover	E value	Per. Ident	Acc. Len	Accession
☑	chr13		2.100e+05	1.545e+06	50%	0.0	99.33%	887524	Query_10986
☑	chr18		69653	1.037e+06	33%	0.0	98.98%	702471	Query_10987

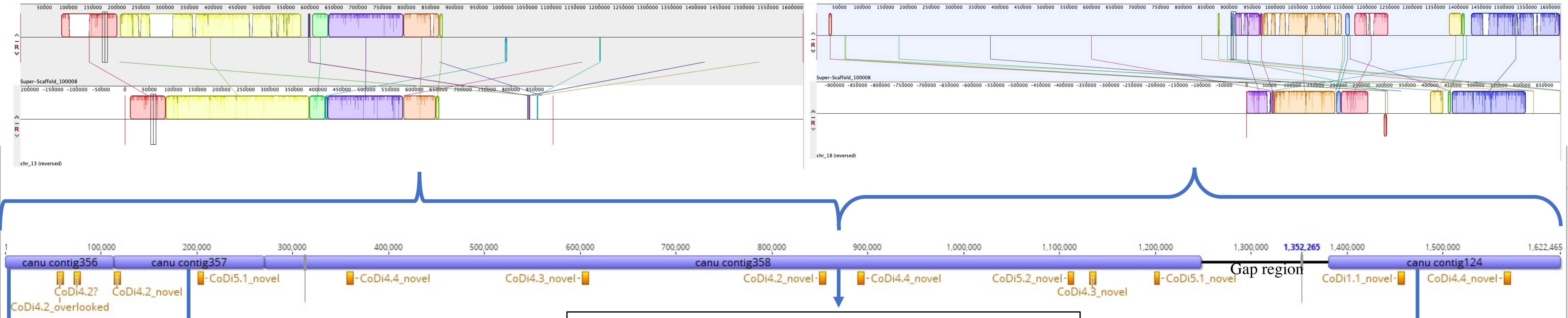

Canu contig357 blasts to chr13 along almost its entire length

	Description	Max Score	Total Score	Query Cover	E value	Per. Ident	Acc. Len
☑	chr13Rx	1.340e+05	2.780e+05	92%	0.0	99.07%	887524
☑	chr18Rx	1046	2188	1%	0.0	91.76%	702471

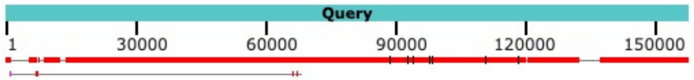

Portions of Canu contig358 blast to chr13 and chr18. Raw read mapping does not support contig358 as a misassembled contig.

	Description	Max Score	Total Score	Query Cover	E value	Per. Ident	Acc. Len	Accession
☑	chr13	2.100e+05	1.159e+06	62%	0.0	99.33%	887524	Query_59720
☑	chr18	69653	5.924e+05	31%	0.0	98.98%	702471	Query_59721

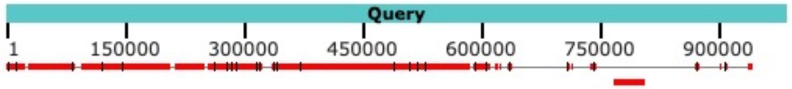

The majority of Canu contig124 blasts strongly to chr18 and aligns to only to the CoDi4.4 locus of chr13

	Description	Common Name	Max Score	Total Score	Query Cover	E value	Per. Ident	Acc. Len	Accession
☑	chr18		58637	4.145e+05	87%	0.0	98.80%	702471	Query_759
☑	chr13		9132	18155	4%	0.0	93.86%	887524	Query_758

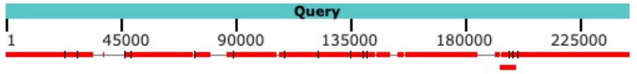

Canu contig356 strongly identifies a portion of the contig to chr13 with the rest of the contig not well resolved to another reference chromosome

	Description	Max Score	Total Score	Query Cover	E value	Per. Ident	Acc. Len
☑	chr13Rx	49358	89450	45%	0.0	98.51%	887524
☑	chr18Rx	8257	28322	14%	0.0	98.47%	702471

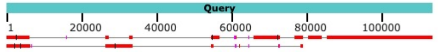

Supplementary Figure 8E

Super-Scaffold_100005

chr2—chr12 hybrid

Reference chr2 and chr12 blasted against Super-Scaffold_100005

	Description	Max Score	Total Score	Query Cover	E value	Per. Ident	Acc. Len	Accession
☑	chr2	2.607e+05	2.718e+06	58%	0.0	99.53%	1497954	Query_29820
☑	chr12	2.274e+05	1.654e+06	32%	0.0	99.37%	901853	Query_29821

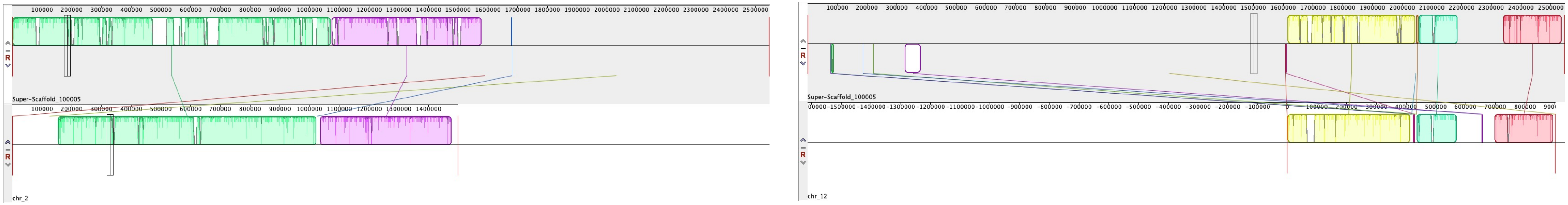

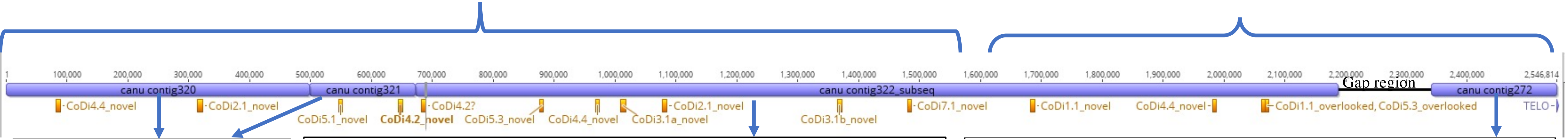

Canu contig320 and contig321 have 99% identity to chr2 with a qcov against chr2 of 91% and 84%, respectively. Both contigs have <10% qcov to chr12

Portions of Canu contig322 blast to chr2 and chr12 but raw read mapping does not indicate contig322 as a misassembled contig and does not support splitting this contig.

	Description	Max Score	Total Score	Query Cover	E value	Per. Ident	Acc. Len	Accession
☑	chr2	2.607e+05	1.614e+06	58%	0.0	99.53%	1497954	Query_53562
☑	chr12	2.274e+05	1.187e+06	38%	0.0	99.37%	901853	Query_53563

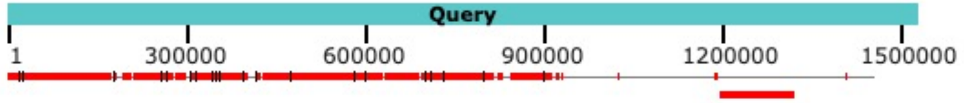

Canu contig272 does not align to chr2 but does show strong identity along its entire length to chr12.

Description	Max Score	Total Score	Query Cover	E value	Per. Ident	Acc. Len	Accession
chr12	1.096e+05	3.577e+05	94%	0.0	99.33%	901853	Query_21987
chr2	1164	6143	1%	0.0	96.85%	1497954	Query_21986

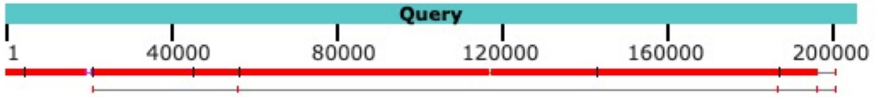

Supplementary Figure 8F

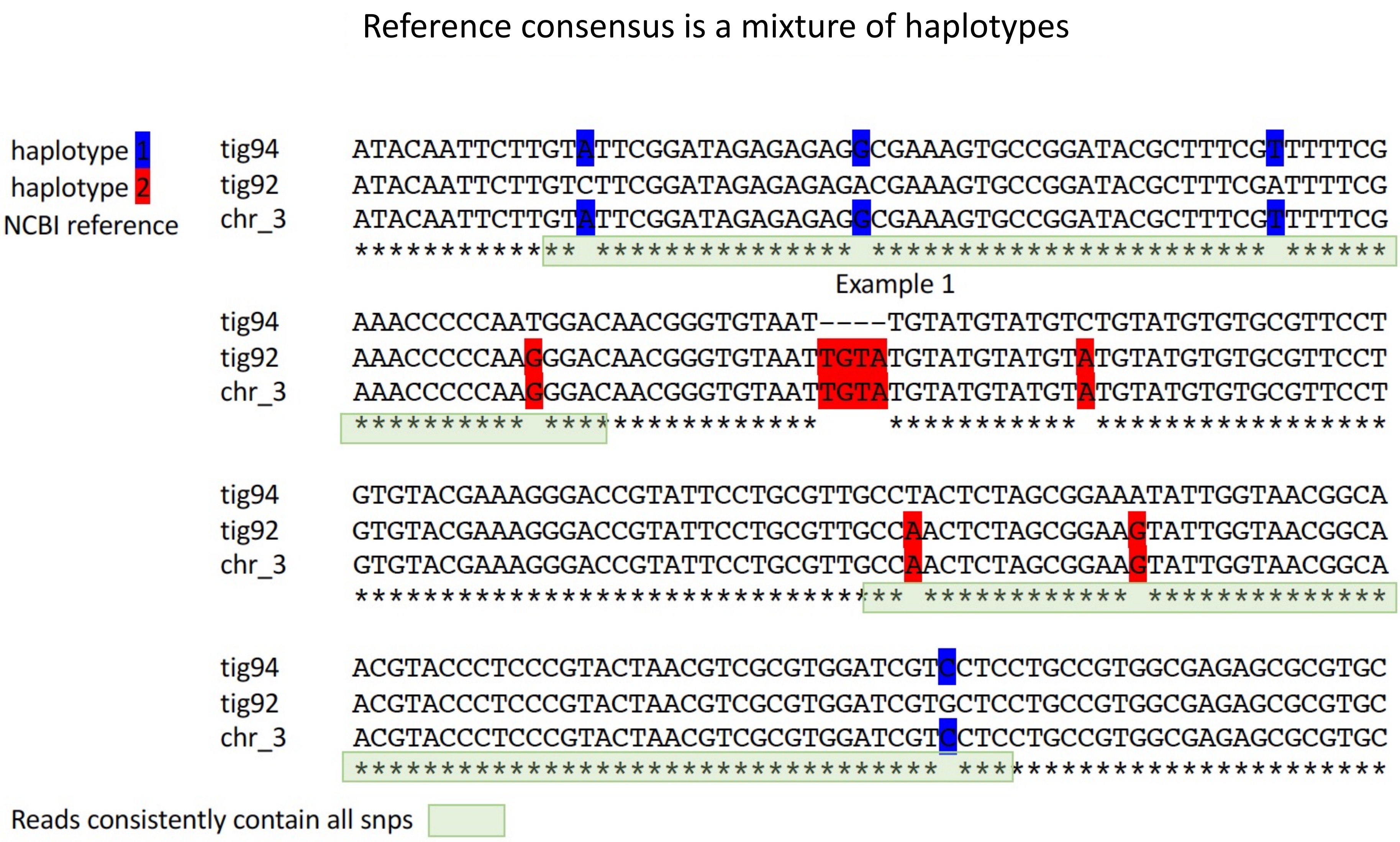

Supplementary Figure 9

Reference consensus is a mixture of haplotypes

IGV visual multiple alignment of Illumina reads –example 1

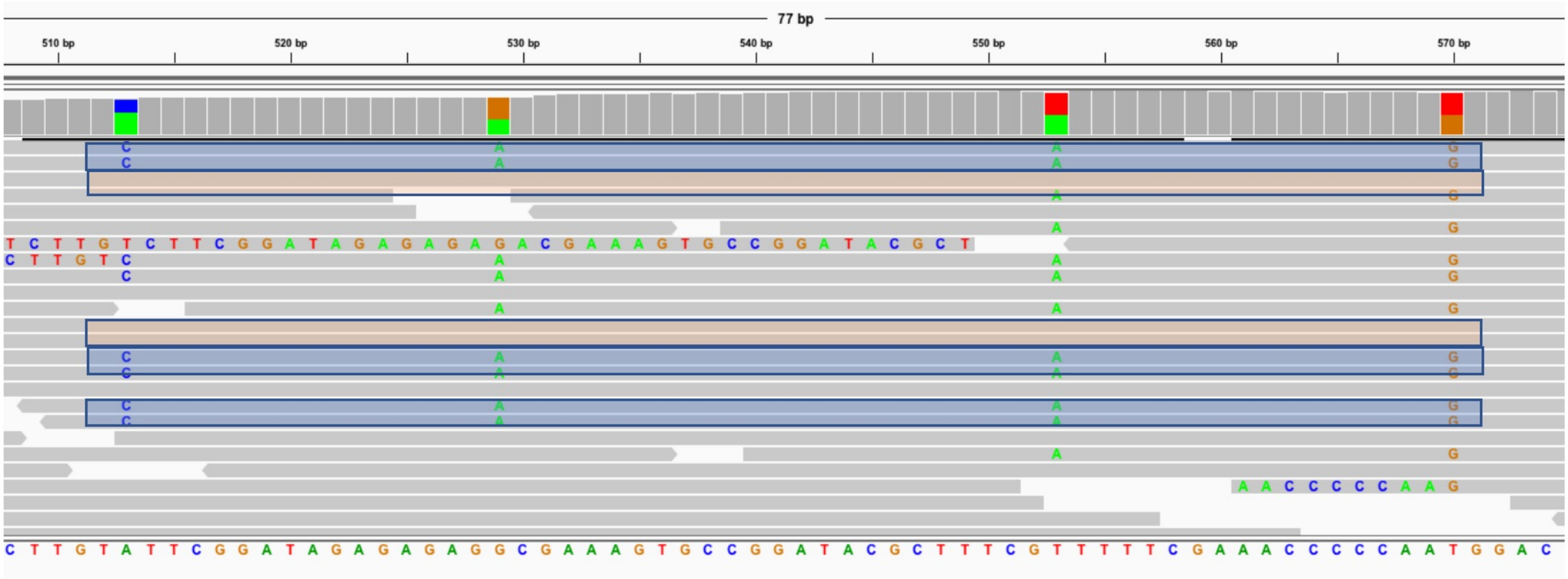

32 reads with haplotype 1

30 reads with haplotype 2

Supplementary Figure 10

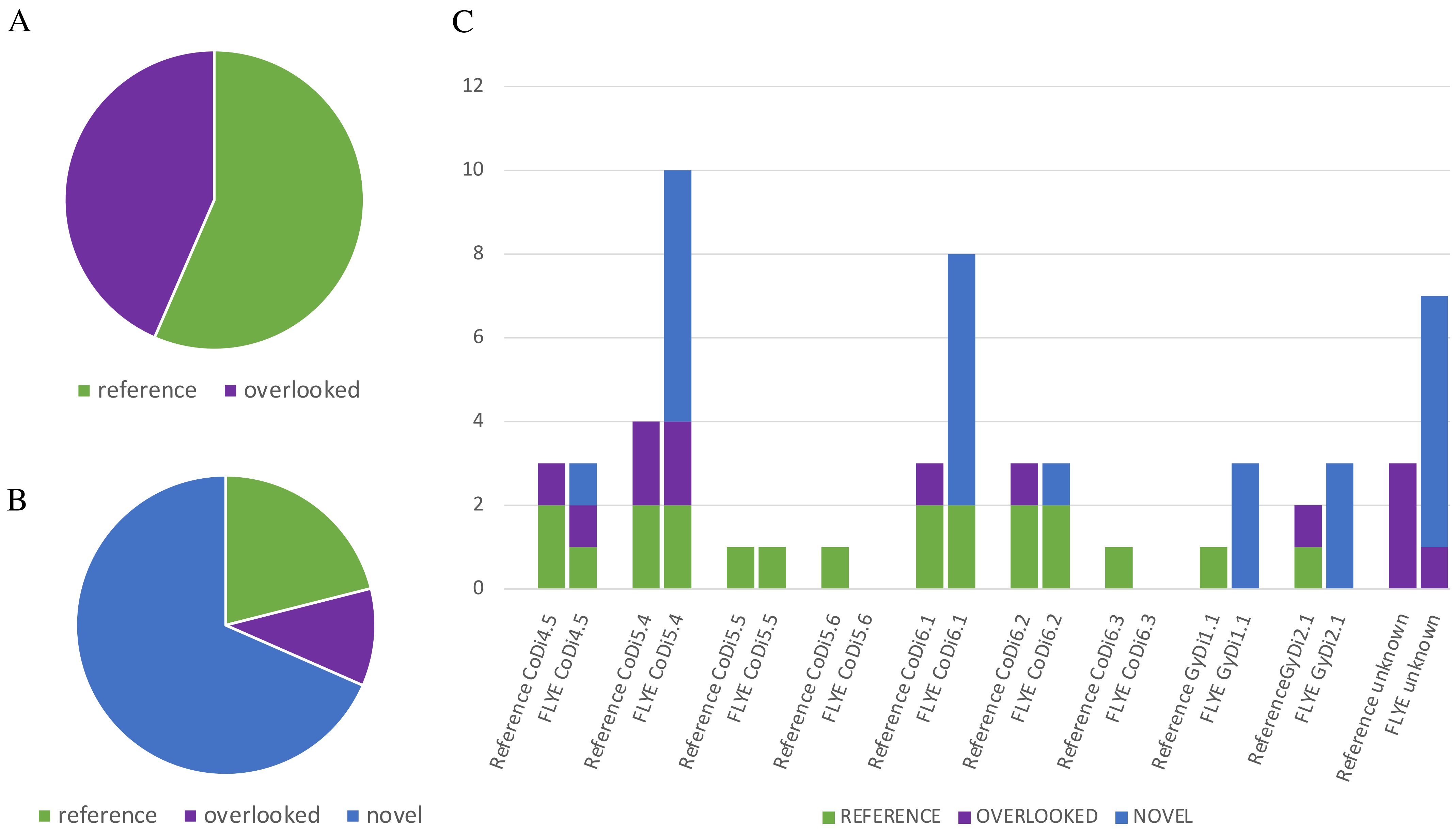

Supplementary Figure 11
